# Development and validation of QMortality risk prediction algorithm to estimate short term risk of death and assess frailty: cohort study

**DOI:** 10.1136/bmj.j4208

**Published:** 2017-09-21

**Authors:** Julia Hippisley-Cox, Carol Coupland

**Affiliations:** Division of Primary Care, University Park, University of Nottingham, Nottingham NG2 7RD, UK

## Abstract

**Objectives** To derive and validate a risk prediction equation to estimate the short term risk of death, and to develop a classification method for frailty based on risk of death and risk of unplanned hospital admission.

**Design** Prospective open cohort study.

**Participants** Routinely collected data from 1436 general practices contributing data to QResearch in England between 2012 and 2016. 1079 practices were used to develop the scores and a separate set of 357 practices to validate the scores. 1.47 million patients aged 65-100 years were in the derivation cohort and 0.50 million patients in the validation cohort.

**Methods** Cox proportional hazards models in the derivation cohort were used to derive separate risk equations in men and women for evaluation of the risk of death at one year. Risk factors considered were age, sex, ethnicity, deprivation, smoking status, alcohol intake, body mass index, medical conditions, specific drugs, social factors, and results of recent investigations. Measures of calibration and discrimination were determined in the validation cohort for men and women separately and for each age and ethnic group. The new mortality equation was used in conjunction with the existing QAdmissions equation (which predicts risk of unplanned hospital admission) to classify patients into frailty groups.

**Main outcome measure** The primary outcome was all cause mortality.

**Results** During follow-up 180 132 deaths were identified in the derivation cohort arising from 4.39 million person years of observation. The final model included terms for age, body mass index, Townsend score, ethnic group, smoking status, alcohol intake, unplanned hospital admissions in the past 12 months, atrial fibrillation, antipsychotics, cancer, asthma or chronic obstructive pulmonary disease, living in a care home, congestive heart failure, corticosteroids, cardiovascular disease, dementia, epilepsy, learning disability, leg ulcer, chronic liver disease or pancreatitis, Parkinson’s disease, poor mobility, rheumatoid arthritis, chronic kidney disease, type 1 diabetes, type 2 diabetes, venous thromboembolism, anaemia, abnormal liver function test result, high platelet count, visited doctor in the past year with either appetite loss, unexpected weight loss, or breathlessness. The model had good calibration and high levels of explained variation and discrimination. In women, the equation explained 55.6% of the variation in time to death (R^2^), and had very good discrimination—the D statistic was 2.29, and Harrell’s C statistic value was 0.85. The corresponding values for men were 53.1%, 2.18, and 0.84. By combining predicted risks of mortality and unplanned hospital admissions, 2.7% of patients (n=13 665) were classified as severely frail, 9.4% (n=46 770) as moderately frail, 43.1% (n=215 253) as mildly frail, and 44.8% (n=223 790) as fit.

**Conclusions** We have developed new equations to predict the short term risk of death in men and women aged 65 or more, taking account of demographic, social, and clinical variables. The equations had good performance on a separate validation cohort. The QMortality equations can be used in conjunction with the QAdmissions equations, to classify patients into four frailty groups (known as QFrailty categories) to enable patients to be identified for further assessment or interventions.

## Introduction

NHS England (the commissioning body for the English National Health Service) recently announced that from July 2017 all general practices in England will be contractually obliged to identify patients with moderate and severe frailty as part of the new General Medical Service contract. This is particularly challenging because frailty is a relatively new concept that does not have an agreed definition. Current approaches to defining frailty involve identifying patients with a collection of diagnoses, symptoms, and social factors.^1^ ^2^ These factors may be combined into a frailty score. This score is then used to identify patients at risk of important or preventable outcomes such as unplanned hospital admissions or death in the near future.

Although recent guidance from the National Institute for Health Care and Excellence on multiple morbidities^3^ has recommended tools to predict risk of unplanned hospital admissions,^4^ ^5^ NICE was unable to identify any equations to reliably predict all cause mortality. NICE identified 41 studies that validated an equation to predict all cause mortality, all of which had major limitations. For example, some equations had been developed for purposes other than to predict all cause mortality.^2^ Other limitations were omitting key determinants of death, such as age and sex; giving equal weighting to all component factors within an equation (for example, wearing glasses could have equal weighting to ischaemic heart disease); using small unrepresentative samples; inappropriate handling of missing data; and poor reporting and poor performance of the tool in predicting death. The NICE guideline therefore recommended that research should be undertaken to develop new robust equations to identify patients with reduced life expectancy so that relevant assessments and interventions can be targeted appropriately.

We aimed to address the NICE research recommendation by developing a new equation to predict risk of death over a one year period among people aged 65 and older using a large validated medical research database of representative patients in primary care. Our secondary objective was to develop a definition of frailty directly based on risk of outcomes. Instead of creating a frailty index in the hope that it would predict unplanned admissions and all cause mortality, we decided to work the other way round. Starting with principled estimators of unplanned admissions and all cause mortality, we decided to develop a new classification of frailty, known as QFrailty. This would group people into four categories—severely frail, moderately frail, mildly frail, or fit—based on their absolute risks of an unplanned hospital admission or death within a year. This could then provide an outcomes based classification to improve on the electronic frailty index recommended by NHS England.^2^

## Methods

### Study design and data source

We undertook a cohort study in a large population of primary care patients in England who were registered with practices contributing to the QResearch database (version 42). All practices had to have used EMIS computer system for at least a year. We randomly allocated three quarters of the practices to the derivation dataset and the remainder to a validation dataset. We identified an open cohort of patients aged 65-100 years registered with practices between 1 January 2012 and 30 September 2016. Exclusions were patients who did not have a valid National Health Service number and those who did not have a postcode related Townsend score (eg, patients had moved to newly built houses with new postcodes not yet linked to deprivation data or patients were homeless or did not have a permanent residence). We determined an entry date to the cohort for each patient, which was the latest of his or her 65th birthday, date of registration with the practice plus one year, date on which the practice computer system was installed plus one year, and beginning of the study period (1 January 2012). Patients were censored at the earliest date of death, de-registration with the practice, last upload of computerised data, or the study end date (30 September 2016).

### Outcomes

Our primary outcome was all cause mortality, using the date of death recorded on the QResearch database. We chose to evaluate risk of death at one year for comparability with other studies and to meet the requirements of the research recommendation in the NICE guidelines. The QResearch database is linked at individual patient level to the hospital admissions data and to mortality records obtained from the Office for National Statistics. The records are linked using a project specific pseudonymised NHS number. The recording of NHS numbers is valid and complete for 99.8% of QResearch patients, 99.9% for ONS mortality records, and 98% for hospital admissions records.^4^ ^6^

### Predictor variables

We examined several predictor variables (see box 1) based on established risk factors already included in the QAdmissions equation^4^ (which predicts risk of unplanned hospital admissions) and variables highlighted in the related literature.^2^ ^3^ ^7^ ^8^ ^9^ ^10^

**Box 1 Predictor variables**

- Age (continuous variable)
- Geographical region in England (10 regions)
- Townsend deprivation score. This is an area level continuous score based on the patients’ postcode.^11^ Originally developed by Townsend,^11^ the score includes unemployment (as a percentage of those aged 16 or more who are economically active), non-car ownership (as a percentage of all households), non-home ownership (as a percentage of all households), and household overcrowding. These variables are measured for a given area of approximately 120 households, through the 2011 census, and combined to give a Townsend score for that area. A higher Townsend score implies a greater level of deprivation
- Ethnic group (nine categories)
- Alcohol intake (<1 unit/day, 1-2 units/day, 3-6 units/day, 7-9 units/day, ≥9 units/day) (see www.nhs.uk/Livewell/alcohol/Pages/alcohol-units.aspx)
- Smoking status (non-smoker; former smoker; light, moderate, or heavy smoker)
- Body mass index (continuous variable)
- Unplanned admissions in past 12 months (0, 1, 2, or ≥3) as recorded on the linked hospital data
- Poor mobility (poor mobility, housebound, confined to chair, bedridden, requires home visit, receives mobility allowance)
- Lives in a care home (nursing home or residential care)
- Lives alone
- Atrial fibrillation
- Congestive heart failure
- Cardiovascular disease (myocardial infarction, angina, stroke, or transient ischaemic attack)
- Valvular heart disease
- Peripheral vascular disease
- Treated hypertension (hypertension and current antihypertensive treatment)
- Chronic kidney disease (stages 4 or 5)
- Diabetes (none, type 1, type 2)
- Hypothyroidism
- Hyperthyroidism
- Cancer
- Chronic liver disease or pancreatitis
- Malabsorption (including Crohn’s disease, ulcerative colitis, coeliac disease, steatorrhea, blind loop syndrome)
- Peptic ulcer (gastric or duodenal ulcer, simple or complicated ulcer)
- Asthma or chronic obstructive airways disease
- Epilepsy
- Dementia
- Learning disability
- Osteoporosis
- Fragility fracture (hip, spine, shoulder, or wrist fracture)
- Parkinson’s disease or syndrome
- Rheumatoid arthritis
- Falls
- Bipolar disorder or schizophrenia
- Depression in past 12 months
- Venous thromboembolism
- Anaemia (haemoglobin <110 g/L)
- Abnormal liver function test result (bilirubin, alanine aminotransferase, or γ glutamyltransferase more than three times the upper limit of normal)
- High platelet count (>480×10^9^/L)
- Leg ulcer (leg, shin, ankle or foot ulcer, ischaemic neuropathic, arterial, or venous ulcer)
- Blindness (registered blind or partially sighted or visual impairment)
- Appetite loss in past 12 months
- Weight loss in past 12 months (unexplained or abnormal weight loss)
- Urinary incontinence in past 12 months
- Nocturia in past 12 months
- Urinary retention in past 12 months (acute or chronic retention)
- Syncope (vasovagal symptom, faint, collapse, “funny turn,” drop attack) in past 12 months
- Dizziness in past 12 months
- Insomnia in past 12 months
- Dyspnoea in past 12 months (breathless at rest or on exertion, paroxysmal nocturnal dyspnoea)
- Hearing impairment or deafness in past 12 months
- Loneliness in past 12 months
- Use of anticoagulants (≥2 prescriptions in past six months)
- Use of antidepressants (≥2 prescriptions in past six months)
- Use of antipsychotics (≥2 prescriptions in past six months)
- Use of corticosteroids (≥2 prescriptions in past six months)
- Non-steroidal anti-inflammatory drugs (≥2 prescriptions in past six months)

The number of unplanned hospital admissions in the previous 12 months was derived from information on the linked hospital records. All predictor variables were based on the latest coded information recorded in the general practice record before entry to the cohort.

### Derivation and validation of the models

We developed and validated the risk prediction equations using established methods.^10^ ^12^ ^13^ ^14^ ^15^ To replace missing values for body mass index, smoking status, and alcohol intake we used multiple imputation with chained equations and used these values in our main analyses.^16^ ^17^ ^18^ ^19^ We carried out five imputations as this has relatively high efficiency^20^ and was a pragmatic approach accounting for the size of the datasets and capacity of the available servers and software. We included all predictor variables in the imputation model, along with age interaction terms, the Nelson-Aalen estimator of the baseline cumulative hazard, and the outcome indicator.

Cox’s proportional hazards models estimated the coefficients for each risk factor in men and women separately. We used Rubin’s rules to combine the results across the imputed datasets.^21^ We used fractional polynomials^22^ to model non-linear risk relations with continuous variables (age and body mass index) using data from patients with recorded values to derive the fractional polynomial terms. Initially we fitted full models. We retained variables if they had an adjusted hazard ratio of <0.90 or >1.10 (for binary variables) and were statistically significant at the 0.01 level. We examined interactions between predictor variables and age at study entry and included statistically significant interactions in the final models.

For each variable from the final model we used the regression coefficients as weights, which we combined with the baseline survivor function at one year to derive risk equations.^23^ We estimated the baseline survivor function based on zero values of centred continuous variables, with all binary predictor values set to zero.

### Validation of the models

In the validation cohort we used multiple imputation to replace missing values for body mass index, smoking status, and alcohol intake. Five imputations were done. We applied the risk equations for men and women obtained from the derivation cohort to the validation cohort and calculated measures of discrimination. As in previous studies,^24^ we calculated R^2^ values (explained variation where higher values indicate a greater proportion of variation in survival time explained by the model^25^), D statistic^26^ (a measure of discrimination that quantifies the separation in survival between patients with different levels of predicted risk, where higher values indicate better discrimination), and Harrell’s C statistic at one year and combined these across datasets using Rubin’s rules. Harrell’s C statistic^27^ is a measure of discrimination (separation) that quantifies the extent to which those with earlier events have higher risk scores. It is similar to the receiver operating characteristic statistic but takes account of the censored nature of the data. Higher values of Harrell’s C indicate better performance of the model for predicting the relevant outcome. A value of 1 indicates the model has perfect discrimination. A value of 0.5 indicates that the model discrimination is no better than chance. We also evaluated these performance measures in five age groups and nine ethnic groups. We calculated 95% confidence intervals for the performance statistics to allow comparisons with alternative models for the same outcome and across different subgroups.^28^

We assessed calibration of the mortality score by comparing the mean predicted risks evaluated at one year with the observed risks by 10th of predicted risk. The observed risks were obtained using the Kaplan-Meier estimates evaluated at one year for men and women. We also evaluated performance by calculating Harrell’s C statistics in individual general practices and combined the results using meta-analytical techniques.^29^

We also applied the latest version of the QAdmissions score to the validation cohort and calculated measures of discrimination for unplanned hospital admissions over one year.

### Decision curve analysis

We used decision curve analysis in the validation cohort to evaluate the net benefits of the mortality score.^30^ ^31^ ^32^ This method assesses the benefits of correctly identifying people who will have an event compared with the harms from a false positive classification (which could, for example, lead to unnecessary distress or interventions). The net benefit of a risk equation at a given risk threshold is given by calculating the difference between the proportion of true positives and the proportion of false positives multiplied by the odds of the risk threshold. We calculated the net benefits across a range of threshold probabilities and compared these with alternative strategies of “intervention in everyone” and “intervention in no one.” In general, the strategy with the highest net benefit at any given risk threshold is considered to have the most clinical value.

### Development of frailty categories

Since there is no currently accepted threshold for classifying high risk of death, we examined the distribution of predicted risks and calculated a series of centile values. For each centile threshold, we calculated the sensitivity, specificity, and positive and negative predictive values of death over a one year follow-up period. Using the latest version of the QAdmissions score we also examined the distribution of patients by their risk of unplanned hospital admission over one year.^4^ We identified unplanned admissions using the hospital episode statistics linked to QResearch as in the original paper.^4^ We then classified patients into four frailty groups based on a combination of their predicted risk of unplanned admission and their predicted risk of death over the next 12 months such that the proportion of patients in each group was broadly similar to that published elsewhere for the “electronic frailty score” (EFI) based on a similar English population.^2^ In the internal validation of the EFI score, 3% of the validation cohort were categorised as having severe frailty, 12% as having moderate frailty, 35% as having mild frailty, and 50% as fit. The corresponding values for the EFI external validation cohort were 4%, 16%, 37%, and 43%.

We repeated some analyses, restricting the validation cohort to those with two or more medical conditions who would meet the NICE broad definition of having multiple morbidities. The supplementary tables present the results. To maximise the power and also generalisability of the results we used all the relevant patients on the database. STATA (version 14) was used for all analyses. We adhered to the TRIPOD statement for reporting.^33^

### Patient involvement

No patients were involved in setting the research question or the outcome measures, nor were they involved in the design or implementation of the study. Patient representatives from the QResearch advisory board have written the information for patients on the QResearch website about the use of the database for research. They have also advised on dissemination of the results, including the use of lay summaries describing the research and its results.

## Results

### Overall study population

*Derivation cohort*—overall, 1436 QResearch practices in England met our inclusion criteria, of which 1079 were randomly assigned to the derivation dataset, with the remainder (n=357) assigned to the validation cohort. We identified 1 471 558 patients in the derivation cohort aged 65-100 years. Of these, we excluded 2550 (0.2%) who did not have a valid NHS number and a further 2410 (0.2%) who did not have a recorded Townsend score, leaving 1 466 598 for the derivation analysis.

*Validation cohort*—we identified 500 816 patients in the validation cohort aged 65-100 years. Of those, we excluded 505 (0.1%) who did not have a valid NHS number and a further 833 (0.2%) who did not have a recorded Townsend score, leaving 499 478 for the validation analysis.

### Baseline characteristics

Table 1 shows the baseline characteristics of men and women in the derivation and validation cohorts. In the derivation cohort, self assigned ethnicity was recorded in 80.3% (n=1 177 596), smoking status in 99.0% (n=1 451 343), alcohol intake in 92.0% (n=1 349 728), and body mass index in 90.2% (n=1 322 929). Overall, 86.8% (n=1 273 310) had complete data for smoking status, alcohol intake, and body mass index. The mean age was 75.3 years, and 12.0% (n=175 915) of patients had one or more unplanned hospital admissions in the past 12 months, 42.8% (n=628 106) had treated hypertension, 21.0% (n=307 499) had cardiovascular disease, 15.1% (n=220 886) had type 2 diabetes, 18.8% (n=276 001) were prescribed antidepressants, and 18.3% (n=268 821) were prescribed non-steroidal anti-inflammatory drugs (NSAIDs). The corresponding results for the validation cohort were similar.

**Table 1 tbl1:** Baseline characteristics of patients aged 65-100 years in derivation and validation cohorts. Values are numbers (percentages) unless stated otherwise

Characteristics	Derivation cohort (n=1 466 598)	Validation cohort (n=499 478)
Men	661 694 (45.1)	224 547 (45.0)
Mean (SD) age (years)	75.3 (8.0)	75.3 (8.0)
Mean (SD) Townsend deprivation score*	−0.7 (3.0)	−0.6 (2.9)
Age band (years):		
65-69	442 386 (30.2)	151 236 (30.3)
70-74	323 574 (22.1)	110 885 (22.2)
75-79	267 641 (18.2)	90 415 (18.1)
80-84	208 967 (14.2)	70 640 (14.1)
85-100	224 030 (15.3)	76 302 (15.3)
Region in England:		
East Midlands	81 431 (5.6)	31 707 (6.3)
East of England	112 915 (7.7)	27 419 (5.5)
London	189 005 (12.9)	69 316 (13.9)
North east	61 255 (4.2)	19 346 (3.9)
North west	256 504 (17.5)	83 381 (16.7)
South central	165 438 (11.3)	81 030 (16.2)
South east	148 212 (10.1)	36 796 (7.4)
South west	179 601 (12.2)	75 051 (15.0)
West Midlands	187 298 (12.8)	50 884 (10.2)
Yorkshire and Humber	84 939 (5.8)	24 548 (4.9)
Ethnic group:		
Recorded	1 177 596 (80.3)	391 503 (78.4)
White or not recorded	1 387 476 (94.6)	474 518 (95.0)
Indian	18 601 (1.3)	6065 (1.2)
Pakistani	8008 (0.5)	3160 (0.6)
Bangladeshi	5449 (0.4)	1516 (0.3)
Other Asian	8268 (0.6)	2731 (0.5)
Caribbean	15 916 (1.1)	4415 (0.9)
Black African	7953 (0.5)	2196 (0.4)
Chinese	2957 (0.2)	907 (0.2)
Other ethnic group	11 970 (0.8)	3970 (0.8)
Smoking status:		
Recorded	1 451 343 (99.0)	494 576 (99.0)
Non-smoker	785 482 (53.6)	264 165 (52.9)
Former smoker	520 517 (35.5)	179 517 (35.9)
Light smoker	83 757 (5.7)	28 549 (5.7)
Moderate smoker	38 971 (2.7)	13 918 (2.8)
Heavy smoker	22 616 (1.5)	8427 (1.7)
Alcohol intake (units/day):		
Recorded	1 349 728 (92.0)	456 150 (91.3)
Non-drinker	539 314 (36.8)	181 050 (36.2)
<1	416 894 (28.4)	141 499 (28.3)
1-2	182 700 (12.5)	62 769 (12.6)
3-6	191 013 (13.0)	63 658 (12.7)
7-9	13 455 (0.9)	4820 (1.0)
>9	5403 (0.4)	2045 (0.4)
Unplanned hospital admissions in past 12 months:		
None	1 290 683 (88.0)	439 774 (88.0)
1	116 749 (8.0)	39 834 (8.0)
2	35 430 (2.4)	11 884 (2.4)
≥3	23 736 (1.6)	7986 (1.6)
Clinical and social characteristics:		
Poor mobility	142 924 (9.7)	55 134 (11.0)
Living in a residential or nursing home	27 471 (1.9)	9275 (1.9)
Atrial fibrillation	120 671 (8.2)	40 729 (8.2)
Any cancer	162 406 (11.1)	55 175 (11.0)
Asthma or chronic obstructive airways disease	227 226 (15.5)	76 470 (15.3)
Congestive heart failure	57 572 (3.9)	19 749 (4.0)
Cardiovascular disease	307 499 (21.0)	104 925 (21.0)
Treated hypertension	628 106 (42.8)	215 567 (43.2)
Hyperthyroidism	23 432 (1.6)	7672 (1.5)
Hypothyroidism	134 476 (9.2)	46 172 (9.2)
Chronic kidney disease	25 641 (1.7)	8877 (1.8)
Type 1 diabetes	10 123 (0.7)	3348 (0.7)
Type 2 diabetes	220 886 (15.1)	73 909 (14.8)
Venous thromboembolism	59 066 (4.0)	20 275 (4.1)
Epilepsy	21 773 (1.5)	7519 (1.5)
Leg ulcer	41 654 (2.8)	13 957 (2.8)
Chronic liver disease or pancreatitis	13 380 (0.9)	4587 (0.9)
Depression in the past 12 months	114 669 (7.8)	37 749 (7.6)
Malabsorption (Crohn’s disease, ulcerative colitis, coeliac disease)	19 178 (1.3)	6455 (1.3)
Parkinson’s disease	16 101 (1.1)	5308 (1.1)
Peptic ulcer disease	92 236 (6.3)	30 992 (6.2)
Fragility fracture	4020 (0.3)	1015 (0.2)
Osteoporosis	100 931 (6.9)	34 071 (6.8)
Rheumatoid arthritis	30 628 (2.1)	10 312 (2.1)
Learning disability	15 836 (1.1)	4982 (1.0)
Dementia	65 828 (4.5)	22 710 (4.5)
Bipolar disorder or schizophrenia	13 137 (0.9)	4261 (0.9)
Hearing loss	247 928 (16.9)	81 544 (16.3)
Cataract	204 807 (14.0)	68 571 (13.7)
Registered blind or impaired vision	30 987 (2.1)	11 209 (2.2)
Falls	205 672 (14.0)	69 669 (13.9)
≥2 medical conditions (see above)	862 019 (58.8)	292 096 (58.5)
Current prescribed drugs:		
Anticoagulants	92 484 (6.3)	31 443 (6.3)
Antidepressants	276 001 (18.8)	96 122 (19.2)
Antipsychotics	25 661 (1.7)	8785 (1.8)
Corticosteroids	136 214 (9.3)	46 332 (9.3)
Non-steroidal anti-inflammatory drugs	268 821 (18.3)	89 246 (17.9)
Symptoms or situation recorded by doctor in past 12 months:		
Appetite loss	3549 (0.2)	1106 (0.2)
Unexplained weight loss	10 710 (0.7)	3541 (0.7)
Dyspnoea	104 563 (7.1)	36 316 (7.3)
Syncope	16 008 (1.1)	5445 (1.1)
Urinary incontinence	15 022 (1.0)	4845 (1.0)
Urinary retention	4935 (0.3)	1633 (0.3)
Nocturia	7268 (0.5)	2517 (0.5)
Dizziness	48 891 (3.3)	16 777 (3.4)
Insomnia	18 187 (1.2)	6033 (1.2)
Loneliness	1144 (0.1)	244 (0.0)
Lives alone	8030 (0.5)	2718 (0.5)
Clinical values:		
Body mass index recorded	1 322 929 (90.2)	448 322 (89.8)
Mean (SD) body mass index	27.3 (4.9)	27.3 (5.0)
Full blood count recorded	1 091 839 (74.4)	376 829 (75.4)
Platelet count recorded	1 269 085 (86.5)	432 388 (86.6)
Liver function test result recorded	1 172 729 (80.0)	384 423 (77.0)
Complete data†	873 765 (59.6)	285 655 (57.2)
Complete data‡	1 273 310 (86.8)	429 123 (85.9)
Anaemia (haemoglobin <110 g/L)	46 766 (3.2)	16 085 (3.2)
Abnormal liver function test result§	29 409 (2.0)	9928 (2.0)
High platelet count	14 010 (1.0)	4753 (1.0)

*This is an area level continuous score based on patients’ postcode. A higher Townsend score implies a greater level of deprivation.

†Complete data for all of smoking status, alcohol intake, body mass index, haemoglobin concentration, platelet count, and liver function test before cohort entry.

‡Complete data for imputed variables: smoking status, alcohol intake, and body mass index.

§γ glutamyltransferase, alanine aminotransferase, or bilirubin levels more than three times the upper limit of normal.

Supplementary table 1 shows the patients’ number of medical conditions. In the derivation cohort, 17.3% (n=253 585) did not have any of the 29 conditions listed, 23.9% (n=350 994) had one condition, and 58.8% (n=862 019) had two or more conditions.

### Incidence of death

Table 2 shows the number of patients who died during the study period, the person years of follow-up, and the death rates by age and sex. Overall in the derivation cohort, 180 132 deaths arose from 4.39 million person years of follow-up. In the validation cohort, 61 446 deaths arose from 1.49 million person years of follow-up. In the derivation and validation cohorts 581 702 and 197 834 people, respectively, had five or more years of follow-up.

**Table 2 tbl2:** Number of deaths, person years of follow up, and death rate per 1000 person years of observation (95% confidence intervals) in derivation and validation cohort

Characteristics	Derivation cohort				Validation cohort		
	No of deaths	Person years	Death rate per 1000 person years (95% CI)		No of deaths	Person years	Death rate per 1000 person years (95% CI)
Women	94 999	2 405 772	39.5 (39.2 to 39.7)		32 726	821 029	39.9 (39.4 to 40.3)
Men	85 133	1 981 380	43.0 (42.7 to 43.3)		28 720	672 665	42.7 (42.2 to 43.2)
Age band (years):							
65-69	17 288	1 410 337	12.3 (12.1 to 12.4)		6023	483 057	12.5 (12.2 to 12.8)
70-74	21 599	1 022 097	21.1 (20.9 to 21.4)		7408	350 038	21.2 (20.7 to 21.7)
75-79	29 141	829 908	35.1 (34.7 to 35.5)		9895	280 968	35.2 (34.5 to 35.9)
80-84	37 679	605 479	62.2 (61.6 to 62.9)		12 611	203 123	62.1 (61.0 to 63.2)
85-89	37 694	342 402	110.1 (109.0 to 111.2)		12 849	115 905	110.9 (109.0 to 112.8)
90-94	27 061	145 561	185.9 (183.7 to 188.1)		9278	49 511	187.4 (183.6 to 191.2)
95-100	9,670	31 368	308.3 (302.2 to 314.5)		3382	11 092	304.9 (294.8 to 315.3)
Overall	180 132	4 387 152	41.1 (40.9 to 41.2)		61 446	1 493 694	41.1 (40.8 to 41.5)

### Predictor variables

Table 3 shows the adjusted hazard ratios for the final models for women and men in the derivation cohort. The final model included the variables: fractional polynomial terms for age, fractional polynomial terms for body mass index, Townsend score, ethnic group, smoking status, alcohol intake, unplanned hospital admissions in the past 12 months, atrial fibrillation, antipsychotics, cancer, asthma or chronic obstructive pulmonary disease, living in a care home, congestive heart failure, corticosteroids, cardiovascular disease, dementia, epilepsy, learning disability, leg ulcer, chronic liver disease or pancreatitis, Parkinson’s disease, poor mobility, rheumatoid arthritis, chronic kidney disease, type 1 diabetes, type 2 diabetes, venous thromboembolism, anaemia, abnormal liver function test result, high platelet count, and visits to a general practitioner in the past 12 months with either appetite loss, unexplained weight loss, or dyspnoea (breathlessness). The other variables tested did not meet the criteria for inclusion in the final model.

**Table 3 tbl3:** Adjusted hazard ratios (95% confidence interval) for death in derivation cohort. Models also include fractional polynomial terms for age and body mass index

Characteristics	Adjusted hazard ratio (95% CI)	
	Women	Men
Townsend score (5 unit increase)*	1.13 (1.11 to 1.15)	1.2 (1.18 to 1.21)
Ethnic group:		
White or not recorded	1.00	1.00
Indian	0.89 (0.83 to 0.96)	0.78 (0.73 to 0.83)
Pakistani	0.99 (0.89 to 1.10)	0.77 (0.70 to 0.85)
Bangladeshi	0.92 (0.81 to 1.04)	0.79 (0.71 to 0.87)
Other Asian	0.82 (0.72 to 0.92)	0.67 (0.59 to 0.75)
Caribbean	0.75 (0.80 to 0.81)	0.71 (0.66 to 0.77)
Black African	0.68 (0.59 to 0.78)	0.71 (0.63 to 0.80)
Chinese	0.53 (0.42 to 0.68)	0.64 (0.53 to 0.78)
Other ethnic group	0.87 (0.80 to 0.95)	0.75 (0.69 to 0.82)
Smoking status:		
Non-smoker	1.00	1.00
Former smoker	1.18 (1.16 to 1.20)	1.18 (1.16 to 1.20)
Light smoker	1.79 (1.74 to 1.84)	1.71 (1.66 to 1.76)
Moderate smoker	2.07 (1.99 to 2.15)	1.90 (1.82 to 1.98)
Heavy smoker	2.29 (2.17 to 2.41)	2.14 (2.05 to 2.24)
Alcohol intake (units/day):		
Non-drinker	1.00	1.00
<1	0.84 (0.83 to 0.86)	0.85 (0.83 to 0.86)
1-2	0.82 (0.80 to 0.84)	0.82 (0.80 to 0.84)
3-6	0.86 (0.84 to 0.89)	0.86 (0.84 to 0.88)
7-9	1.21 (1.05 to 1.40)	1.12 (1.06 to 1.18)
>9	1.46 (1.22 to 1.76)	1.17 (1.08 to 1.27)
Unplanned admissions in past 12 months:		
None	1.00	1.00
1*	2.05 (1.99 to 2.11)	1.99 (1.94 to 2.04)
2*	2.98 (2.85 to 3.11)	2.87 (2.76 to 2.98)
≥3*	4.69 (4.48 to 4.91)	4.22 (4.06 to 4.40)
Clinical and social characteristics :		
Atrial fibrillation	1.39 (1.37 to 1.42)	1.28 (1.26 to 1.30)
Cancer*	1.91 (1.86 to 1.96)	2.05 (2.00 to 2.09)
Asthma or chronic obstructive pulmonary disease	1.19 (1.17 to 1.22)	1.15 (1.13 to 1.18)
Lives in care home*	1.80 (1.71 to 1.89)	1.61 (1.52 to 1.71)
Congestive heart failure*	1.66 (1.60 to 1.72)	1.74 (1.69 to 1.79)
Cardiovascular disease*	1.31 (1.28 to 1.34)	1.25 (1.22 to 1.27)
Dementia*	2.61 (2.52 to 2.71)	2.34 (2.25 to 2.45)
Epilepsy	1.22 (1.17 to 1.28)	1.25 (1.19 to 1.30)
Learning disability	1.15 (1.10 to 1.19)	1.22 (1.16 to 1.28)
Leg ulcer*	1.61 (1.55 to 1.68)	1.66 (1.60 to 1.73)
Chronic liver disease or pancreatitis*	1.61 (1.48 to 1.74)	1.48 (1.39 to 1.58)
Parkinson’s disease†	1.81 (1.73 to 1.88)	2.16 (2.05 to 2.28)
Poor mobility*	1.63 (1.59 to 1.67)	1.59 (1.55 to 1.63)
Rheumatoid arthritis	1.29 (1.25 to 1.34)	1.19 (1.13 to 1.24)
Chronic kidney disease	1.97 (1.88 to 2.07)	1.86 (1.78 to 1.95)
Type 1 diabetes	1.37 (1.28 to 1.46)	1.30 (1.22 to 1.38)
Type 2 diabetes*	1.35 (1.31 to 1.38)	1.29 (1.26 to 1.32)
Venous thromboembolism	1.19 (1.17 to 1.23)	1.16 (1.13 to 1.19)
Current prescribed drugs:		
Antipsychotics*	1.61 (1.54 to 1.69)	1.60 (1.52 to 1.68)
Corticosteroids*	1.44 (1.40 to 1.48)	1.57 (1.52 to 1.61)
Clinical values:		
Anaemia (haemoglobin <110 g/L*)	1.93 (1.87 to 2.00)	2.10 (2.03 to 2.18)
Abnormal liver function test result‡	1.61 (1.52 to 1.70)	1.67 (1.61 to 1.75)
High platelet count	1.36 (1.31 to 1.42)	1.38 (1.30 to 1.46)
Symptoms recorded in past 12 months:		
Appetite loss	1.30 (1.21 to 1.39)	1.35 (1.24 to 1.48)
Dyspnoea*	1.33 (1.28 to 1.37)	1.28 (1.25 to 1.32)
Weight loss	1.25 (1.20 to 1.31)	1.24 (1.17 to 1.31)

The model in women includes fractional polynomial terms for age (age^−2^ and age^−2^ln (age)) and body mass index (bmi^−1^ and bmi^−5^) and interactions with age for unplanned admissions, Townsend score, anaemia, abnormal liver function test result, type 2 diabetes, cardiovascular disease, heart failure, cancer, dementia, chronic kidney disease, chronic liver disease or pancreatitis, leg ulcer, poor mobility, care home residence, dyspnoea, corticosteroids, and antipsychotics.

The model in men includes fractional polynomial terms for age (age^3^ and age^3^ln (age)) and BMI (bmi^−2^ and bmi^−2^ln(bmi)) and interactions with age for unplanned admissions, Townsend score, anaemia, abnormal liver function test result, type 2 diabetes, cardiovascular disease, heart failure, cancer, dementia, chronic kidney disease, chronic liver disease or pancreatitis, leg ulcer, poor mobility, care home residence, dyspnoea, corticosteroids, antipsychotics, and Parkinson’s disease.

*Interaction with age, hazard ratios evaluated at mean age for men and women.

†interaction with age, hazard ratios evaluated at mean age for men only.

‡γ glutamyltransferase, alanine aminotransferase, or bilirubin levels more than three times the upper limit of normal.

The graphs in figures 1 and 2[Fig f1 f2] show the adjusted hazard ratios in women and men, respectively, for age interaction terms that were statistically significant (see footnote in table 3). For each of these interactions, hazard ratios for the predictors were higher at younger ages compared with older ages.

**Figure f1:**
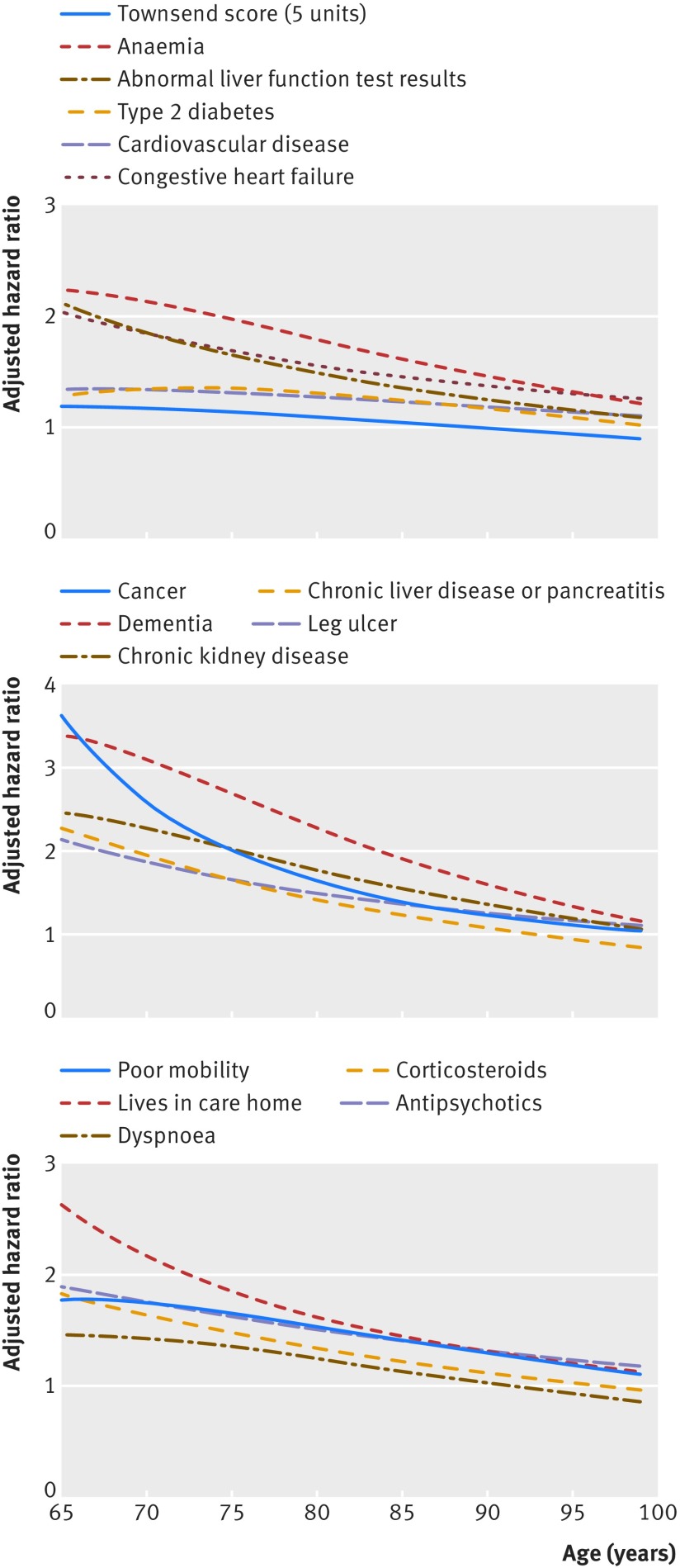
**Fig 1** Hazard ratios for all cause mortality in women by age

**Figure f2:**
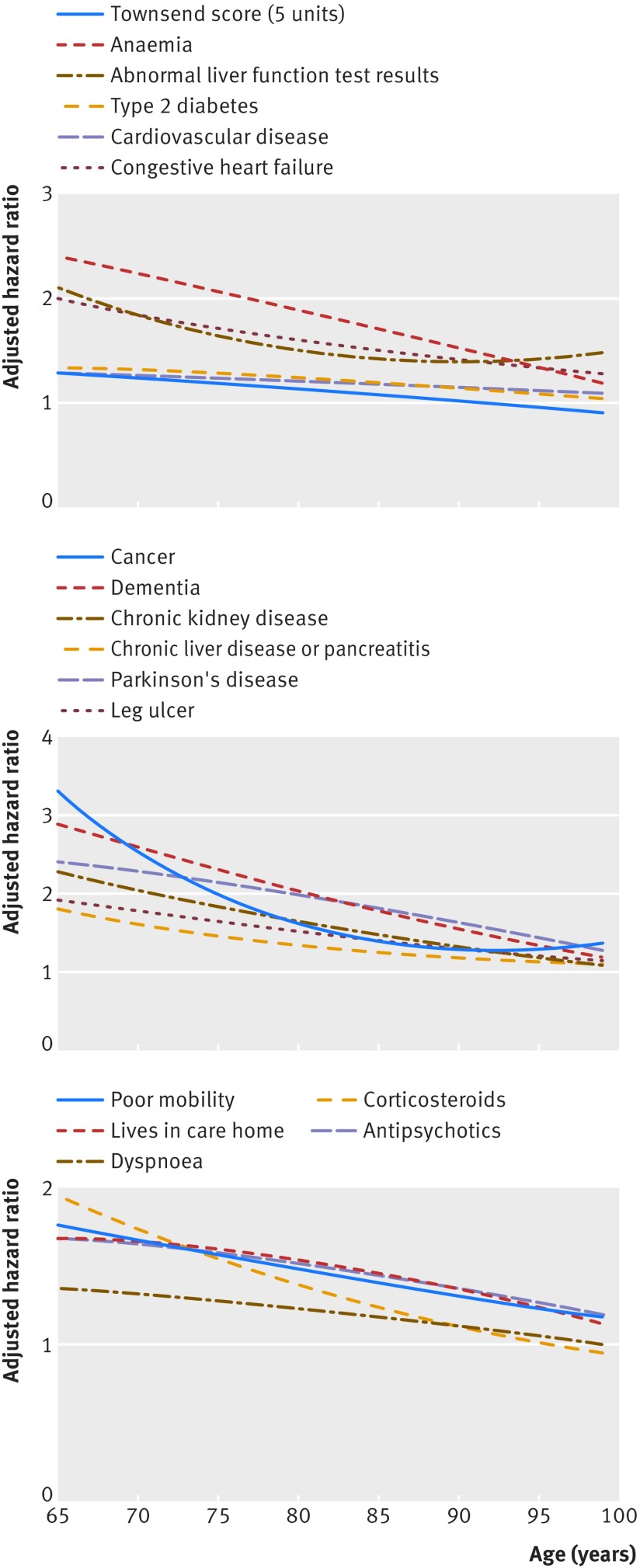
**Fig 2** Hazard ratios for all cause mortality in men by age

### Validation

#### Discrimination

Table 4 shows the performance of the QMortality score in the validation cohort for women and men at one year. Overall, the values for the R^2^, D, and C statistics were higher in women than in men indicating that the score performed better in women than in men. The mortality equation in women explained 55.6% of the variation in time to death (R^2^), the D statistic was 2.29, and Harrell’s C statistic was 0.85. The corresponding values for men were 53.1%, 2.18, and 0.84. Table 4 also shows results for the latest version of the QAdmissions equation for predicting unplanned admissions (based on 160 217 unplanned admissions in the validation cohort over a one year period). Table 4 shows that the performance of the QMortality score for predicting deaths was better than the performance of the QAdmissions score for predicting unplanned admissions.

**Table 4 tbl4:** Performance of QMortality algorithm to predict one year risk of death, and QAdmissions score to predict risk of unplanned admission over one year in men and women aged 65-100 years in validation cohort

Statistics	Mean (95% CI)	
	Women	Men
QMortality score:	
D statistic	2.29 (2.27 to 2.31)	2.18 (2.16 to 2.20)
Harrell’s C	0.853 (0.850 to 0.856)	0.844 (0.841 to 0.847)
R^2^ (%)	55.6 (55.2 to 56.0)	53.1 (52.6 to 53.6)
QAdmissions score:		
D statistic	1.50 (1.49 to 1.51)	1.45 (1.44 to 1.46)
Harrell’s C	0.757 (0.755 to 0.759)	0.751 (0.748 to 0.753)
R^2^ (%)	34.9 (34.5 to 35.2)	33.5 (33.0 to 33.9)

Supplementary table 2 shows the results for the mortality scores by age group and ethnic group. Performance tended to be better in the younger age groups but was similar across all ethnic groups.

Figure 3 shows plots of Harrell’s C statistic for each general practice in the validation cohort against the number of deaths in each practice in women and men separately. The summary (average) C statistic for women was 0.854 (95% confidence interval 0.850 to 0.859) from a random effects meta-analysis. The I^2^ value (ie, the percentage of total variation in C statistic owing to heterogeneity between practices) was 63.2%. The approximate 95% prediction interval for the true C statistic in women in a new practice was 0.80 to 0.91. For men, the summary C statistic was 0.844 (95% confidence interval 0.839 to 0.849). The I^2^ value was 70.3%. The approximate 95% prediction interval for the true C statistic in men in a new practice was 0.76 to 0.92.

**Figure f3:**
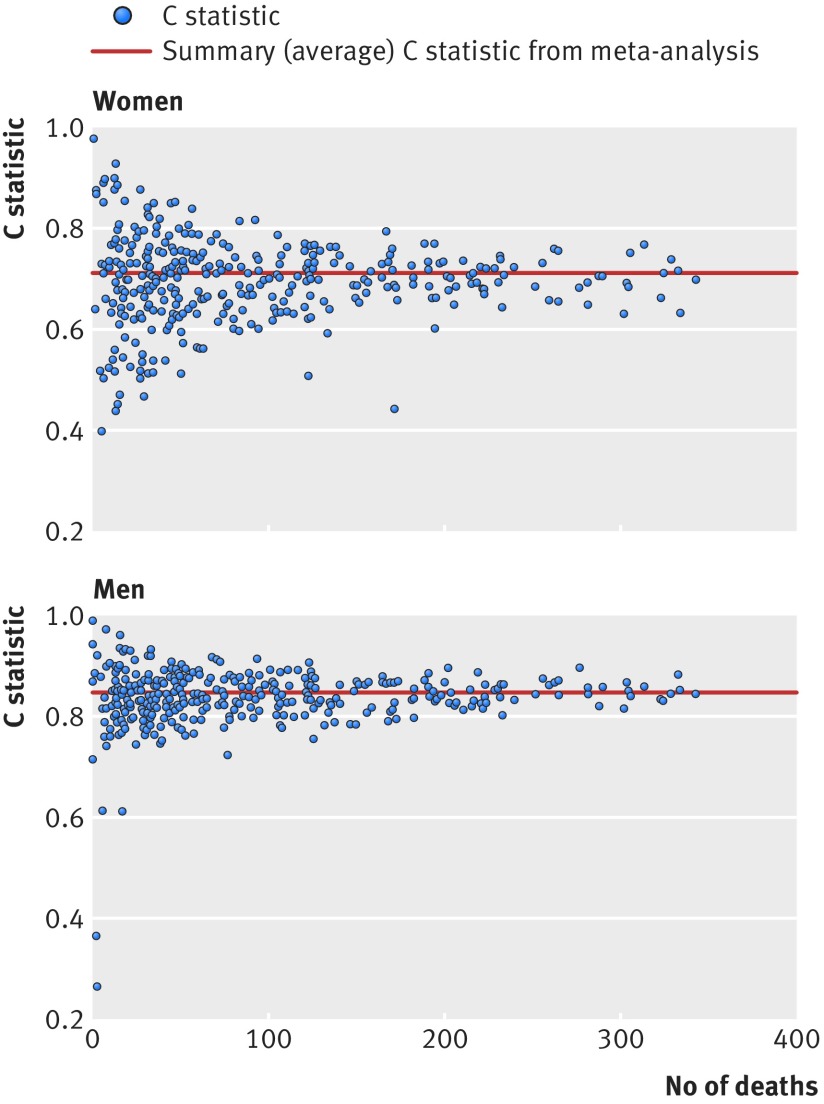
**Fig 3** Plots of discrimination across 357 general practices showing Harrell’s C statistic against number of deaths in women and men

Supplementary table 3 shows the validation statistics for the mortality score among patients with two or more morbidities (as required by the NICE guideline^3^).

#### Calibration

Figure 4 displays the observed risks and mean predicted risks of death across each 10th of the predicted risk score (1 representing the lowest risk and 10 the highest risk). This shows that the equation was well calibrated. Supplementary figure 1a-e shows the calibration within each age group. Supplementary table 5 shows overall calibration by age group and ethnic group and for the top 2%, 10%, and 50% of predicted risk. The results were generally good except for over-prediction in Chinese women and under-prediction in black African women, although numbers of deaths were relatively small in these subgroups.

**Figure f4:**
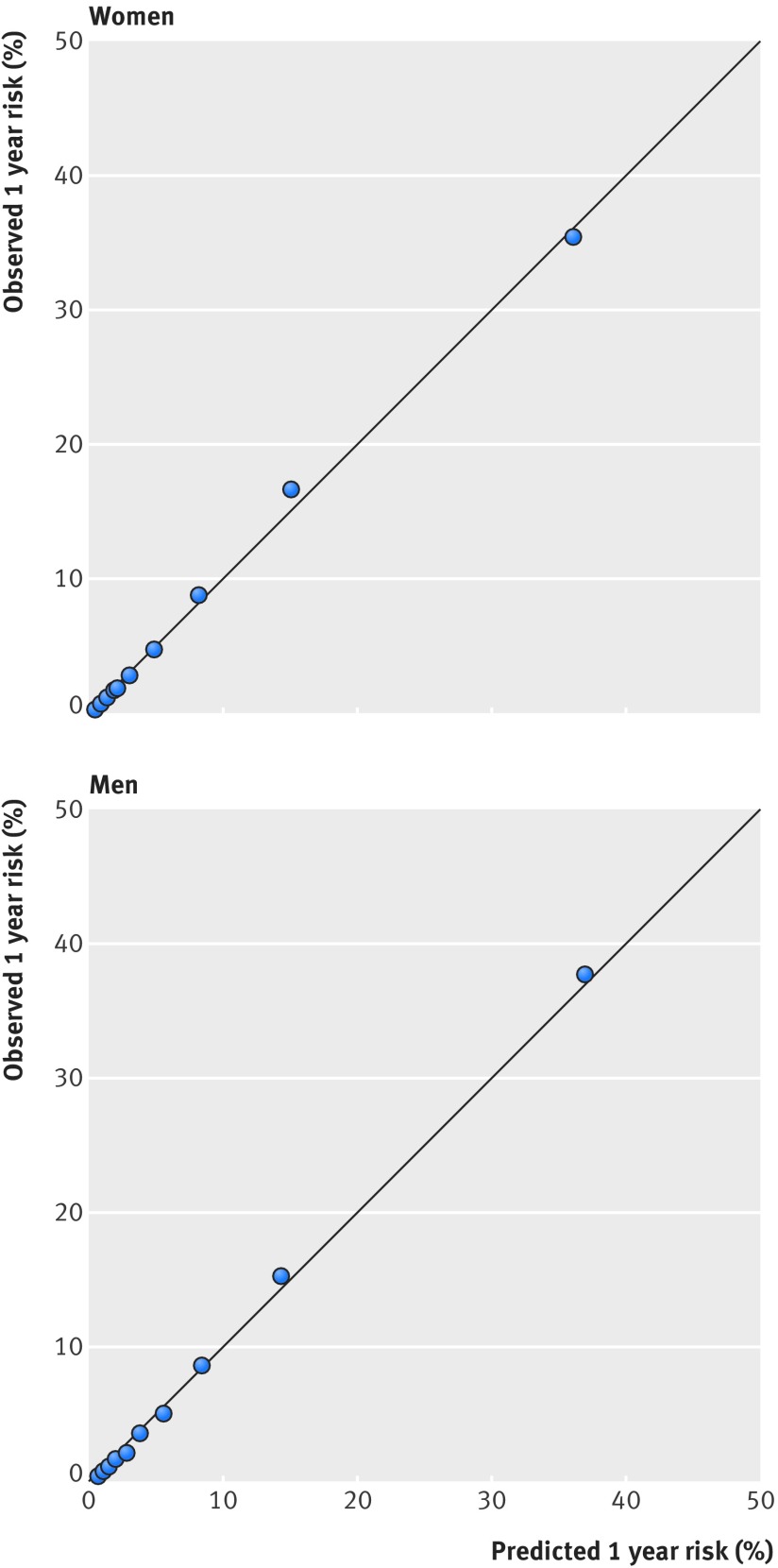
**Fig 4** Predicted and observed risk of all cause mortality at one year in women and men

### Decision curve analysis

Figure 5 displays the net benefit curves for the mortality equations at one year in men and women. These show that the prediction equations had higher net benefit than did strategies based on considering either no patients or all patients for intervention for risk thresholds up to around 50%.

**Figure f5:**
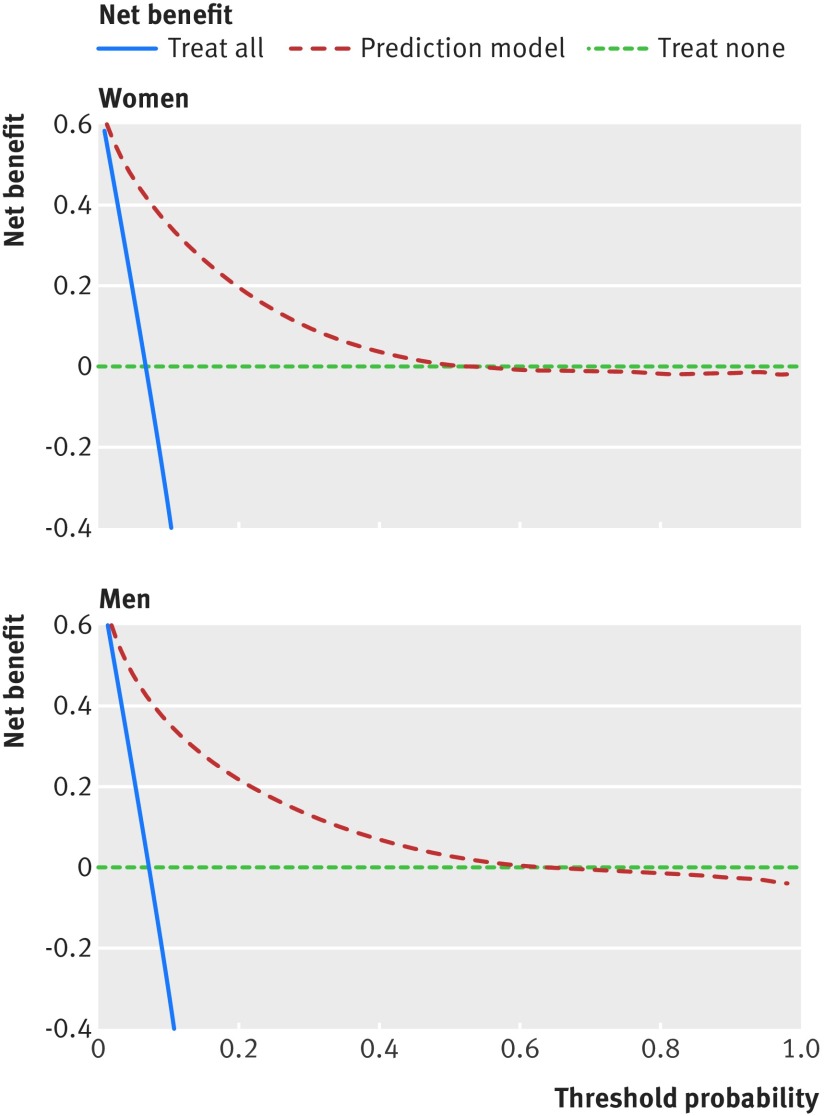
**Fig 5** Decision curve analysis for women and men

#### Sensitivity and specificity

Table 5 shows the sensitivity, specificity, and positive and negative predictive values for the mortality equation at one year for various thresholds based on patients in the validation cohort.

**Table 5 tbl5:** Sensitivity, specificity, and positive and negative predictive values for death at different thresholds of predicted risk of death over one year in validation cohort

Threshold	Risk threshold %	True negative (count)	False negative (count)	False positive (count)	True positive (count)	Sensitivity (%)	Specificity (%)	Positive predictive value (%)	Negative predictive value (%)
Top 1%	58.3	436 007	58 477	2025	2969	4.8	99.5	59.5	88.2
Top 2%	47.0	433 741	55 748	4291	5698	9.3	99.0	57.0	88.6
Top 3%	40.2	431 282	53 212	6750	8234	13.4	98.5	55.0	89.0
Top 4%	35.4	428 786	50 713	9246	10 733	17.5	97.9	53.7	89.4
Top 5%	31.7	426 128	48 377	11 904	13 069	21.3	97.3	52.3	89.8
Top 10%	20.3	411 084	38 447	26 948	22 999	37.4	93.8	46.0	91.4
Top 15%	14.3	393 760	30 797	44 272	30 649	49.9	89.9	40.9	92.7
Top 20%	10.6	374 710	24 873	63 322	36 573	59.5	85.5	36.6	93.8
Top 25%	8.2	354 411	20 198	83 621	41 248	67.1	80.9	33.0	94.6
Top 30%	6.4	333 225	16 410	104 807	45 036	73.3	76.1	30.1	95.3
Top 35%	5.1	311 237	13 424	126 795	48 022	78.2	71.1	27.5	95.9
Top 40%	4.2	288 786	10 901	149 246	50 545	82.3	65.9	25.3	96.4
Top 45%	3.4	265 826	8887	172 206	52 559	85.5	60.7	23.4	96.8
Top 50%	2.9	242 548	7191	195 484	54 255	88.3	55.4	21.7	97.1

Table 6 shows that the risk threshold for the top 2% at highest risk of death in the next year was 47.0%, for the top 10% was 20.3%, and for the top 50% was 2.9%. With a risk threshold of 20.3% over one year to identify the 10% of patients with the highest risk of death, the sensitivity for identifying deaths was 37.4%, specificity 97.3%, positive predictive value 46.0%, and negative predictive value 91.4%. Supplementary table 4 shows the results restricted to patients with two or more medical conditions.

**Table 6 tbl6:** Thresholds for classification of patients into one of four frailty groups

1 year risk of death*	1 year risk of unplanned hospital admission*			
	≥60.7%	34.1-60.6%	10.1-34.0%	<10.1%
≥47.0%	Severe frailty	Severe frailty	Severe frailty	Severe frailty
20.3-46.9%	Severe frailty	Moderate frailty	Moderate frailty	Moderate frailty
2.9-20.2%	Severe frailty	Moderate frailty	Mild frailty	Mild frailty
<2.9%	Severe frailty	Moderate frailty	Mild frailty	Fit

Patients are classified according to the highest risk group. For example, a patient with a one year risk of death of 50% and a one year risk of unplanned admission of 8% would be classified in the severely frail group; a patient with a one year risk of death of 50% or a one year risk of unplanned admission of 62% would be classified in the severely frail group; and a patient with a 2% risk of death and a 5% risk of unplanned admission would be classified as “fit.”

*Risk thresholds correspond to highest 2%, 10%, and 50% absolute risks.

The corresponding thresholds for risk of unplanned hospital admission over one year were 60.7% to identify the top 2%, 34.0% for the top 10%, and 10.0% for the top 50% (results not shown).

### Classification of frailty

Table 7 shows the characteristics of patients from the validation cohort split into four QFrailty groups that are broadly equivalent to the proportion of patients reported to be in the four categories according to the EFI.^2^

**Table 7 tbl7:** Characteristics of patients in validation cohort in each of the four frailty categories based on one year risk of unplanned admission or one year risk of death. Values are numbers (column percentages) unless stated otherwise

Characteristics	Group 1: severe frailty	Group 2: moderate frailty	Group 3: mild frailty	Group 4: fit
No of patients	13 665	46 770	215 253	223 790
Row % of total population	2.74	9.36	43.10	44.80
Men	6142 (44.9)	19 219 (41.1)	104 867 (48.7)	94 319 (42.1)
Mean (SD) age (years)	86.1 (8.4)	85.4 (7.6)	78.2 (6.7)	69.8 (3.9)
Mean (SD) Townsend deprivation score*	0.4 (3.0)	0.0 (3.0)	−0.3 (2.9)	−1.1 (2.7)
Mean (SD) body mass index	26.0 (5.8)	26.3 (5.4)	27.4 (5.2)	27.4 (4.6)
Age band (years):				
65-69	694 (5.1)	2032 (4.3)	26 606 (12.4)	121 904 (54.5)
70-74	884 (6.5)	2849 (6.1)	37 271 (17.3)	69 881 (31.2)
75-79	1316 (9.6)	4882 (10.4)	54 995 (25.5)	29 222 (13.1)
80-84	2201 (16.1)	8468 (18.1)	57 188 (26.6)	2783 (1.2)
≥85	8570 (62.7)	28 539 (61.0)	39 193 (18.2)	0 (0)
Smoking status:				
Non-smoker	6000 (43.9)	23 560 (50.4)	104 320 (48.5)	133 214 (59.5)
Former smoker	5936 (43.4)	18 503 (39.6)	84 077 (39.1)	72 028 (32.2)
Light smoker	934 (6.8)	2686 (5.7)	14 853 (6.9)	10 598 (4.7)
Moderate smoker	447 (3.3)	1189 (2.5)	7325 (3.4)	5238 (2.3)
Heavy smoker	348 (2.5)	832 (1.8)	4678 (2.2)	2712 (1.2)
Alcohol intake (units/day):				
Non-drinker	9468 (69.3)	28 232 (60.4)	96 468 (44.8)	62 754 (28.0)
<1	2486 (18.2)	10 752 (23.0)	62 990 (29.3)	79 192 (35.4)
1-2	817 (6.0)	3924 (8.4)	25 892 (12.0)	38 876 (17.4)
3-6	716 (5.2)	3325 (7.1)	26 157 (12.2)	39 692 (17.7)
7-9	105 (0.8)	347 (0.7)	2524 (1.2)	2331 (1.0)
>9	73 (0.5)	190 (0.4)	1222 (0.6)	945 (0.4)
Previous unplanned admissions in past 12 months:				
None	2888 (21.1)	25 293 (54.1)	189 282 (87.9)	222 311 (99.3)
1	3856 (28.2)	13 040 (27.9)	21 480 (10.0)	1458 (0.7)
2	2763 (20.2)	5280 (11.3)	3820 (1.8)	21 (0.0)
≥3	4158 (30.4)	3157 (6.8)	671 (0.3)	0 (0)
Clinical and social characteristics:				
Poor mobility	8312 (60.8)	18 188 (38.9)	24 622 (11.4)	4012 (1.8)
Living in a residential or nursing home	2474 (18.1)	4419 (9.4)	2283 (1.1)	99 (0.0)
Atrial fibrillation	4864 (35.6)	10 779 (23.0)	21 261 (9.9)	3825 (1.7)
Any cancer	3436 (25.1)	9081 (19.4)	33 040 (15.3)	9618 (4.3)
Asthma or chronic obstructive pulmonary disease	4756 (34.8)	11 697 (25.0)	41 269 (19.2)	18 748 (8.4)
Congestive heart failure	3795 (27.8)	6585 (14.1)	8701 (4.0)	668 (0.3)
Cardiovascular disease	8373 (61.3)	21 867 (46.8)	59 835 (27.8)	14 850 (6.6)
Treated hypertension	6310 (46.2)	23 069 (49.3)	110 750 (51.5)	75 438 (33.7)
Chronic kidney disease	1532 (11.2)	2912 (6.2)	4199 (2.0)	234 (0.1)
Type 1 diabetes	358 (2.6)	697 (1.5)	1901 (0.9)	392 (0.2)
Type 2 diabetes	3925 (28.7)	10 350 (22.1)	41 731 (19.4)	17 903 (8.0)
Venous thromboembolism	1857 (13.6)	4300 (9.2)	10 768 (5.0)	3350 (1.5)
Epilepsy	608 (4.4)	1349 (2.9)	3983 (1.9)	1579 (0.7)
Leg ulcer	2036 (14.9)	4025 (8.6)	6851 (3.2)	1045 (0.5)
Chronic liver disease or pancreatitis	535 (3.9)	954 (2.0)	2511 (1.2)	587 (0.3)
Malabsorption (Crohn’s disease, ulcerative colitis, coeliac disease)	213 (1.6)	714 (1.5)	3107 (1.4)	2421 (1.1)
Parkinson’s disease	850 (6.2)	1487 (3.2)	2549 (1.2)	422 (0.2)
Peptic ulcer disease	2519 (18.4)	5778 (12.4)	16 121 (7.5)	6574 (2.9)
Osteoporosis	2387 (17.5)	6401 (13.7)	16 969 (7.9)	8314 (3.7)
Rheumatoid arthritis	680 (5.0)	1617 (3.5)	5447 (2.5)	2568 (1.1)
Learning disability	803 (5.9)	1507 (3.2)	2110 (1.0)	562 (0.3)
Dementia	4484 (32.8)	9726 (20.8)	8251 (3.8)	249 (0.1)
Bipolar disorder or schizophrenia	306 (2.2)	727 (1.6)	2444 (1.1)	784 (0.4)
Registered blind or impaired vision	1287 (9.4)	3148 (6.7)	5422 (2.5)	1352 (0.6)
Falls	6895 (50.5)	16 789 (35.9)	33 768 (15.7)	12 217 (5.5)
Current prescribed drugs:				
Anticoagulants	2526 (18.5)	7238 (15.5)	18 577 (8.6)	3102 (1.4)
Antidepressants	5461 (40.0)	15 267 (32.6)	50 687 (23.5)	24 707 (11.0)
Antipsychotics	1524 (11.2)	2666 (5.7)	3947 (1.8)	648 (0.3)
Corticosteroids	3615 (26.5)	8761 (18.7)	27 963 (13.0)	5993 (2.7)
Non-steroidal anti-inflammatory drugs	717 (5.2)	3636 (7.8)	37 369 (17.4)	47 524 (21.2)
Symptoms recorded in past 12 months:				
Appetite loss	267 (2.0)	362 (0.8)	410 (0.2)	67 (0.0)
Weight loss	564 (4.1)	947 (2.0)	1611 (0.7)	419 (0.2)
Dyspnoea	2724 (19.9)	6811 (14.6)	21 783 (10.1)	4998 (2.2)
Syncope	750 (5.5)	1380 (3.0)	2461 (1.1)	854 (0.4)
Urinary incontinence	550 (4.0)	1047 (2.2)	2314 (1.1)	934 (0.4)
Urinary retention	353 (2.6)	453 (1.0)	670 (0.3)	157 (0.1)
Clinical values:				
Anaemia (haemoglobin <110 g/L)	3399 (24.9)	5322 (11.4)	6572 (3.1)	792 (0.4)
Abnormal liver function test result	998 (7.3)	1853 (4.0)	5559 (2.6)	1518 (0.7)
High platelet count	523 (3.8)	1027 (2.2)	2399 (1.1)	804 (0.4)

*This is an area level continuous score based on patients’ postcode. A higher Townsend score implies a greater level of deprivation.

- Group 1 represents severe frailty. This category includes 13 665 patients (ie, 2.74% of 499 478) who are either in the top 2% at highest risk of death in the next year or in the top 2% at highest risk of unplanned hospital admission.
- Group 2 represents moderate frailty. This category includes 46 770 patients (ie, 9.36% of 499 478) who are either in the top 10% at highest risk of death in the next year or in the top 10% at highest risk of unplanned hospital admission (excluding those in the severe category).
- Group 3 represents mild frailty. This category includes 215 253 patients (ie, 43.1% of 499 478) who are either in the top 50% at highest risk of death in the next year or in the top 50% at highest risk of unplanned hospital admission (excluding those in the severe and moderate categories).
- Group 4 represents being “fit.” This category includes 223 790 patients (ie, 44.80% of 499 478) not in the above three categories.

For example, for those in the severe frailty category, the mean age is 86.1 years, 98.5% (n=13 460) have multimorbidity, 60.8% (n=8312) have poor mobility, 61.3% (n=8373) have cardiovascular disease, 50.5% (n=6895) have had falls, 46.2% (n=6310) have treated hypertension, 40.0% (n=5461) are taking antidepressants, 35.6% (n=4864) have atrial fibrillation, 34.8% (n=4756) have asthma or chronic obstructive pulmonary disease, 32.8% (n=4484) have dementia, 28.7% (n=3925) have type 2 diabetes, 25.1% (n=3436) have a diagnosis of cancer, 24.9% (n=3399) have anaemia, 19.9% (n=2724) have dyspnoea, 18.5% (n=2526) are taking anticoagulants, and 18.4% (n=2519) have peptic ulcer disease.

## Discussion

Recent NICE guidance on multiple morbidities^3^ highlighted the need to develop new robust equations to identify patients in primary care with reduced life expectancy so that relevant assessments and interventions can be targeted appropriately. Existing equations to predict risk of death are based on biased samples, are insufficiently powered, fail to handle missing data appropriately, are poorly reported, or have poor performance to the extent that NICE has been unable to make a positive recommendation for any of the 41 models included in the review.^3^ We therefore developed and validated equations to predict absolute risk of death over the next year in men and women aged 65-100 years. The QMortality equations performed well on a separate validation cohort, with good levels of discrimination and calibration, improving on other equations used to predict all cause mortality.^2^ ^3^ ^34^ The final model has good face validity as it includes demographic and clinical variables that clinicians would expect to affect mortality risk such as age, body mass index, deprivation, ethnicity, smoking status, alcohol intake, unplanned admissions in the past 12 months; atrial fibrillation, antipsychotics, cancer, asthma or chronic obstructive pulmonary disease, living in a care home, congestive heart failure, corticosteroids, cardiovascular disease, dementia, epilepsy, learning disability, leg ulcer, chronic liver disease or pancreatitis, Parkinson’s disease, poor mobility, rheumatoid arthritis, chronic renal disease, type 1 diabetes, type 2 diabetes, venous thromboembolism, anaemia, abnormal liver function test result, high platelet count, and visits to a doctor in the past year with either appetite loss, unexpected weight loss, or breathlessness.

Although the QMortality equation contains many variables, it is intended to be integrated into general practice computer systems where the extraction of data and risk calculation can be automated. We considered whether to develop a more parsimonious model with fewer predictors for use in other clinical settings but decided it would be preferable to have one model and for the user to select default values on the understanding that there may be a degree of under-estimation or over-estimation of risk depending on the predictor in question.

### Potential uses of the frailty classification and mortality index

In this study we have described a specific novel use for mortality estimates, which is to classify patients into four frailty categories. This has been achieved by combining the one year predicted risk of death with the one year predicted risk of unplanned hospital admission to help identify the most severely frail patients for enhanced care packages to meet the immediate requirements of the UK General Medical Services contract. The most severe frailty category will identify patients with particularly high levels of morbidity who are at highest risk of death or unplanned hospital admission. This group of patients is likely to reflect elderly patients who are the most severely frail and who can be identified for focused assessment and intervention as part of the new General Medical Services contract in England. This includes falls assessment and drug review. The QMortality score could be used in conjunction with the QAdmissions score to allocate patients to one of four QFrailty categories. It could also be used recurrently to build and maintain practice based lists of patients with different levels of frailty or mortality risks over time. This could be done as an automated procedure using electronic health records.

The models can also be used in a face-to-face consultation between the patient and clinician with the intention of sharing the information with the patient to assess management options. The decision curve analysis shows there is a higher net benefit for the prediction models than strategies based on considering either no patients or all patients for intervention for risk thresholds up to around 50%. Mortality estimates including cancer stage and grade are already used to help patients with cancer to weigh up the risks and benefits of surgery, chemotherapy, and radiotherapy.^35^ Patients with a high risk of death in the near future may choose to decline aggressive treatments or defer preventive treatments, screening interventions, or interventions for asymptomatic conditions.^36^ Mortality estimates could also be used to help guide the introduction and addition of palliative care to help plan end of life care.^37^ For example, six month mortality estimates in the United States are used to trigger Advance Care Planning and also to determine access to hospice services under the Medicare scheme.^38^ They are also used to improve self awareness of health status; to measure, monitor, and compare outcomes between different healthcare providers^36^; and are used by governments to decrease the burden of certain risk factors at a population level.^34^

### Ethical considerations

We see an important distinction between factors that are included in a risk equation to ensure that the risk estimates are as accurate as possible and how the risk equation is then used in guidelines and clinical practice to ensure ethical, effective, and equitable access to services for everyone. The primary purpose of our paper is to report on the development and validation of new risk equations rather than to produce national policy or clinical guidance, although we recognise the results may be used by policy makers and clinicians. All clinical decisions about the beneficial and safe use of these risk equations necessarily remain the responsibility of the attending clinician. However, there are ethical issues to consider about how the tools might be used. We have analysed this within the “four ethical principles” framework, which is widely used in medical decision making. The four principles are autonomy, beneficence, justice, and non-maleficence.^39^ The new risk equations, when implemented in clinical software, are designed to provide more accurate information for patients and clinicians on which to base decisions, thereby promoting shared decision making and patient autonomy. They are intended to result in clinical benefit by identifying where changes in management are likely to benefit patients, thereby promoting the principle of beneficence. Justice can be achieved by ensuring that the use of the risk equations results in fair and equitable access to health services that are commensurate with the patients’ level of risk. Lastly, the risk assessment must not be used in a way that causes harm either to the individual patient or to others (for example, by introducing or withdrawing treatments where this is not in the patients’ best interest) thereby supporting the non-maleficence principle. How this applies in clinical practice will naturally depend on many factors, especially the patient’s wishes, the evidence base for any interventions, the clinician’s experience, national priorities, and the available resources. The risk assessment equations therefore supplement clinical decision making, not replace it.

### Comparison with the other risk scores

A recent review of 41 mortality risk scores reported in 24 research papers failed to identify any that could be reliably used to predict mortality in a community settitng.^3^ Of the studies reviewed, the Charleston comorbidity index, which consists of 23 variables, achieved the best C statistic, with a value of 0.77 in the internal validation cohort and 0.80 in the external validation cohort.^3^ ^40^ Other studies have used risk scores to predict mortality, such as the John Hopkins Aggregated Diagnostic Groups (ADG) score^41^ and the Hospital-patient One-year Mortality Risk (HOMR) score.^36^ The HOMR score consists of 12 patient variables and eight hospital admission factors and was designed to predict one year mortality risk in adults aged 18-100 years admitted to hospital. It includes fractional polynomial terms for continuous variables and interactions between statistically significant predictors. The HOMR score has excellent calibration and discrimination, with a C statistic of 0.92, although this may reflect the much wider age range in the HOMR study. The ADG score consists of 30 variables and has been validated using a community based sample. However, the C statistic was lower (0.81) than the values for the QMortality score (0.84 in men and 0.85 in women), and the ADG equation is not published or freely available.^41^

The electronic frailty index (EFI) is a simple unweighted count of the number of “deficits” a patient has out of a total of 36, where a deficit is a physical disability or social vulnerability as identified by a consensus panel.^2^ The EFI has also been used to predict mortality in a UK community based population, although performance (based on standard definitions^42^) was poor or fair, with a C statistic of 0.66 on an internal validation cohort and 0.76 on an external validation cohort.^2^ The EFI also had extremely low levels of explained variation in time to death of 0.02-0.04%,^2^ whereas the QMortality scores explained 53% and 55% of the variation in men and women, respectively. The EFI equation has not been published and it does not appear to include continuous variables such as age. The QMortality and QAdmissions equations include all the factors in the EFI, where these predicted either risk of death or risk of unplanned hospital admission. Unlike the EFI, our equations include further key determinants of death and unplanned admissions, such as age, sex, ethnic group, smoking status, alcohol intake, deprivation, and previous unplanned admissions, and also include major conditions—cancer, epilepsy, serious mental illness, chronic liver disease, inflammatory bowel disease, learning disability, specific drug treatments—which are all relevant to risk of outcomes and for which patients are likely to need ongoing careful assessment. Our multivariable analysis has allowed us to attribute appropriate weights to each factor and incorporate interactions between age and different medical conditions. This means, for example, that a patient who is 65 years old with three medical conditions will have a different absolute risk of death or unplanned hospital admission than a patient with the same conditions but who is aged 95 years.

### Methodological considerations

The methods to derive and validate these models are broadly the same as for a range of other clinical risk prediction tools derived from the QResearch database.^7^ ^8^ ^12^ ^43^ ^44^ The strengths and limitations of the approach have already been discussed in detail.^7^ ^14^ ^43^ ^45^ ^46^ ^47^ In summary, key strengths include size, duration of follow-up, representativeness, and lack of selection, recall, and respondent bias. UK general practices have good levels of accuracy and completeness in recording clinical diagnoses and prescribed drugs.^48^ We think our study has good face validity since it has been conducted in the setting where most patients in the UK are assessed, treated, and followed up. Limitations of our study include the lack of formal adjudication of diagnoses, information bias, and potential for bias owing to missing data. Our database has linked hospital admissions data and is therefore likely to have picked up the majority of hospital admissions, thereby minimising ascertainment bias. We focused on two hard outcomes to identify frail patients (unplanned admissions and mortality) rather than admission to a nursing home or decline in function, as both of these are more difficult to measure using electronic health records. Also, for simplicity we grouped all cancers together as a single variable rather than distinguish between different types of cancer and account for grade and stage. This was a pragmatic decision, partly driven by the lack of information in general practice records about grade and stage of cancer and the availability of existing purpose designed tools such as the QCancer prognostic scores.^49^ QMortality will tend to have under-estimated mortality risk in those with a late stage cancer (for example) and over-estimated it in patients with an early stage cancer. We excluded patients without a deprivation score since this group may represent a more transient population where follow-up could be unreliable or unrepresentative. Their deprivation scores are unlikely to be missing at random so we did not think it would be appropriate to impute them.

We have presented sensitivity and specificity values for death at a range of centile values and combined predicted risks of death and unplanned hospital admissions into frailty categories that can be used to identify patients who are most severely frail based on their risk of clinically important outcomes. The present validation has been done on a separate set of practices and individuals to those that were used to develop the score, although the practices all use the same general practice clinical computer system (EMIS, used by 55% of UK general practitioners). An independent validation study would be a more stringent test and should be done, but when such independent studies have examined other risk equations^46^ ^47^ ^50^ ^51^ they have shown similar performance compared with the validation in the QResearch database.^12^ ^43^ ^45^ We have not been able to undertake direct comparisons between the QMortality score and the ADG, EFI, and HOMR scores since these are not publicly available. For transparency, we have published the source code of the QMortality equation on the QAdmissions website (www.qadmissions.org) alongside the QAdmissions equation. The rationale for this is to ensure that those interested in reviewing or using the open source will then be able to find the latest available version as the score continues to be updated. Lastly, our study was not designed to compare the performance of QMortality scores against clinical judgment alone, although we have provided sufficient information to enable other researchers to undertake such a study. Freund et al found that predictive modelling software was more effective at identifying patients at increased risk of hospital admission and death than clinical judgment alone.^52^ However, clinicians may be more effective at identifying those for whom preventive services may have a better impact.^52^ ^53^

### Conclusion

We have developed a new equation to quantify absolute risk of death within the next year in people aged 65 or more, taking account of demographic, social, and clinical variables. The equation provides a valid measure of absolute risk of death in the general population of patients aged 65 or more as shown by the performance in a separate validation cohort. The equation can be used in conjunction with the QAdmissions equation to classify patients into four QFrailty groups to enable their identification for focused assessments and interventions.

**What is already known on this topic**

Recent NICE guidance on multiple morbidities has highlighted the need to develop new robust equations to identify patients in primary care with reduced life expectancy so that relevant assessments and interventions can be targeted appropriately
Existing equations to predict risk of death are based on biased samples, are insufficiently powered, fail to handle missing data appropriately, are poorly reported, or have poor performance to the extent that NICE has been unable to make a positive recommendation on any tool

**What this study adds**

A new equation (QMortality) quantified absolute risk of death within the next one year in people aged 65 or more, taking account of demographic, social, and clinical variables
QMortality provides a valid measure of absolute risk of death in the general population of older patients, as shown by its performance in a separate validation cohort
QMortality can be used in conjunction with the QAdmissions equation for unplanned hospital admissions to classify patients into four QFrailty groups to enable identification for focused assessments and interventions

## Web extra

Extra material supplied by authors

## Notes

A simple web calculator can be used to implement the QMortality algorithm (http://qmortality.org), which will be publicly available alongside the paper. It also has the open source software for download. A web calculator to implement the combined QMortality and QAdmissions calculator to derive the four frailty categories is available here http://qfrailty.org. We acknowledge the contribution of EMIS practices who contribute to the QResearch database and EMIS and the University of Nottingham for expertise in establishing, developing, and supporting the QResearch database. The hospital episodes statistics data used in this analysis are re-used by permission from the NHS Digital who retain the copyright. We thank the Office for National Statistics for providing the mortality data. ONS and NHS Digital bear no responsibility for the analysis or interpretation of the data

Ethical approval: This study was approved by the East Midlands Derby Research Ethics Committee (reference 03/4/021).

Data sharing: The equations presented in this paper will be released as Open Source Software under the GNU lesser GPL v3. The open source software allows use without charge under the terms of the GNU lesser public license version 3. Closed source software can be licensed at a fee.

Transparency: The lead author (JHC) affirms that the manuscript is an honest, accurate, and transparent account of the study being reported; that no important aspects of the study have been omitted; and that any discrepancies from the study as planned (and, if relevant, registered) have been explained.
