# Supplementary material for: Development and validation of QMortality risk prediction algorithm to estimate short term risk of death and assess frailty: cohort study
Source: BMJ. 2017 Sep 21;358:j4208. doi: 10.1136/bmj.j4208 (PMC5606253; doi:10.1136/bmj.j4208)
Supplement: Supplementary file 1 — Supplementary information: Additional information [file hipj039243.ww1.pdf]

Supplementary figures

Figure 1a calibration in patients aged 65-69 years

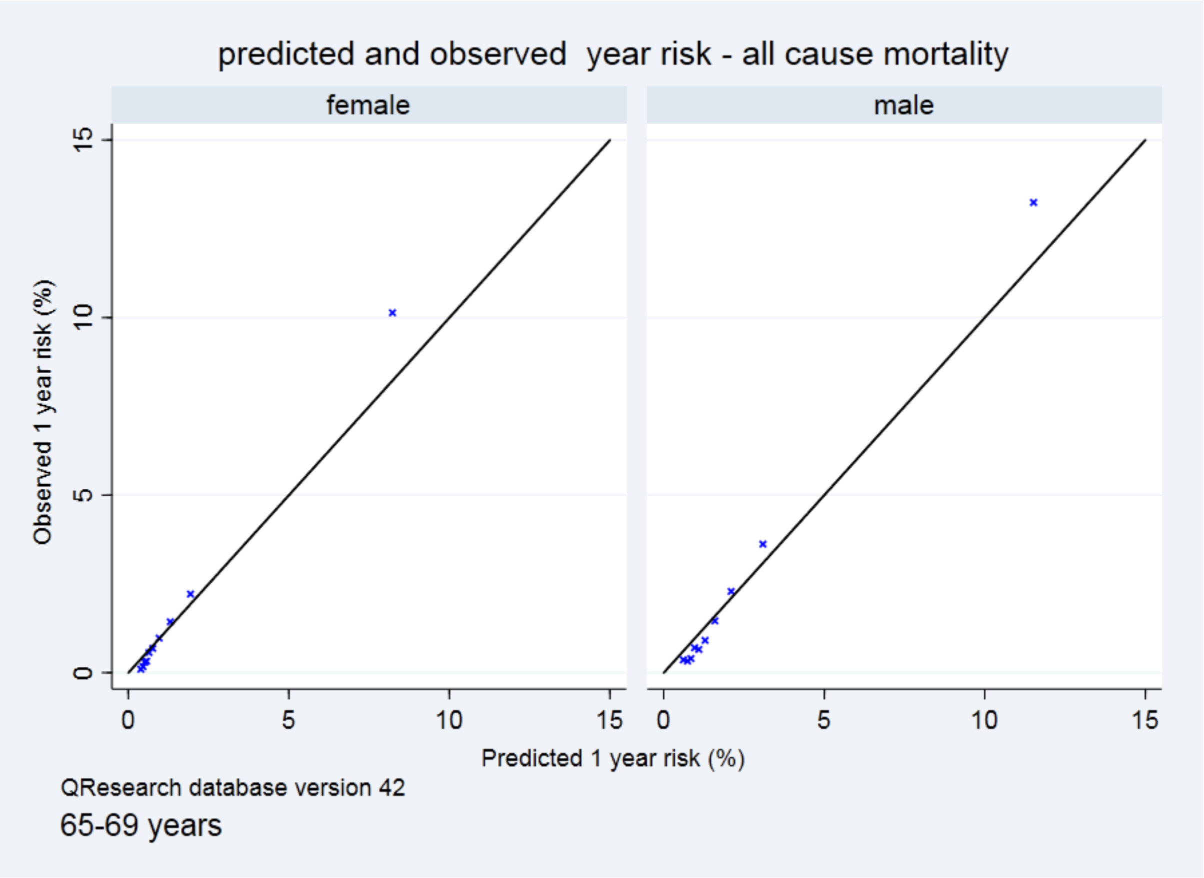

Figure 1b calibration in patients aged 70-74 years

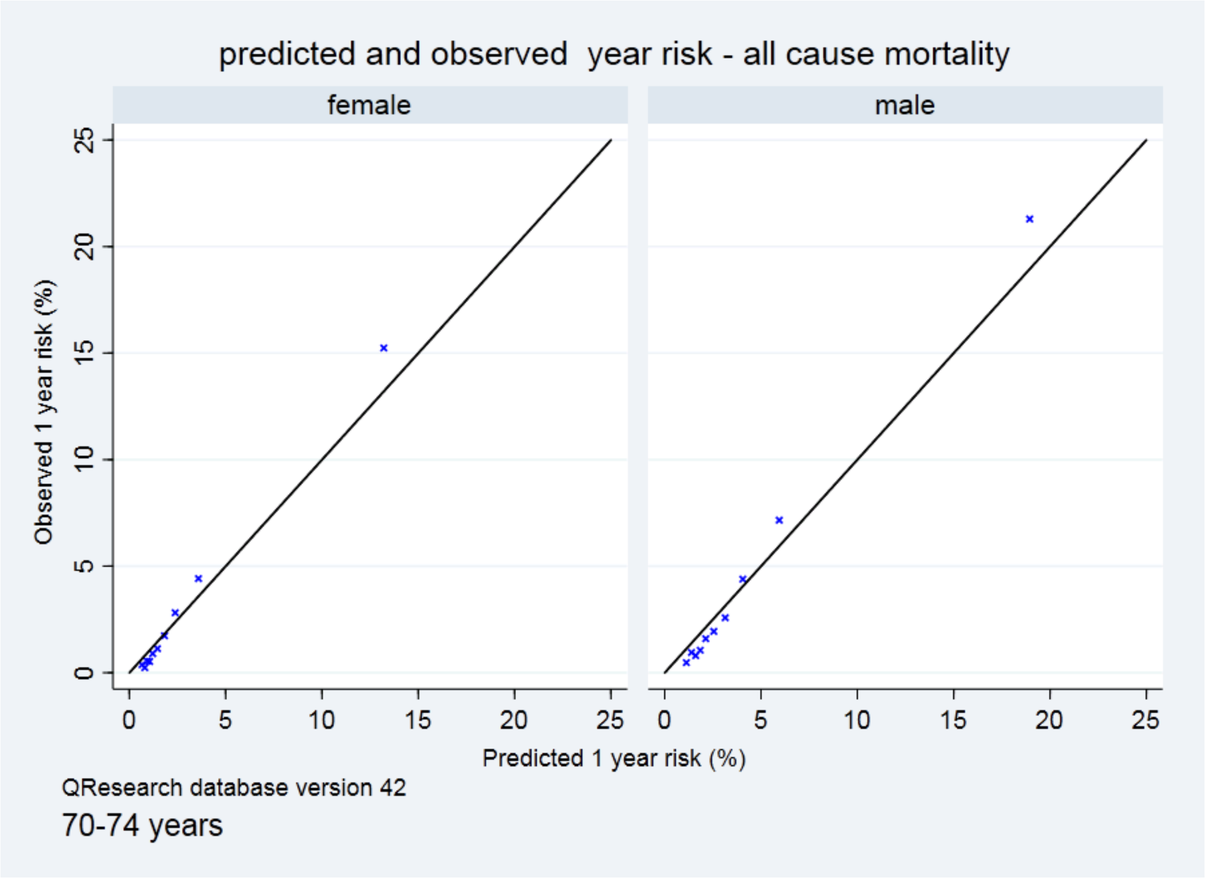

Figure 1c calibration in patients aged 75-79 years

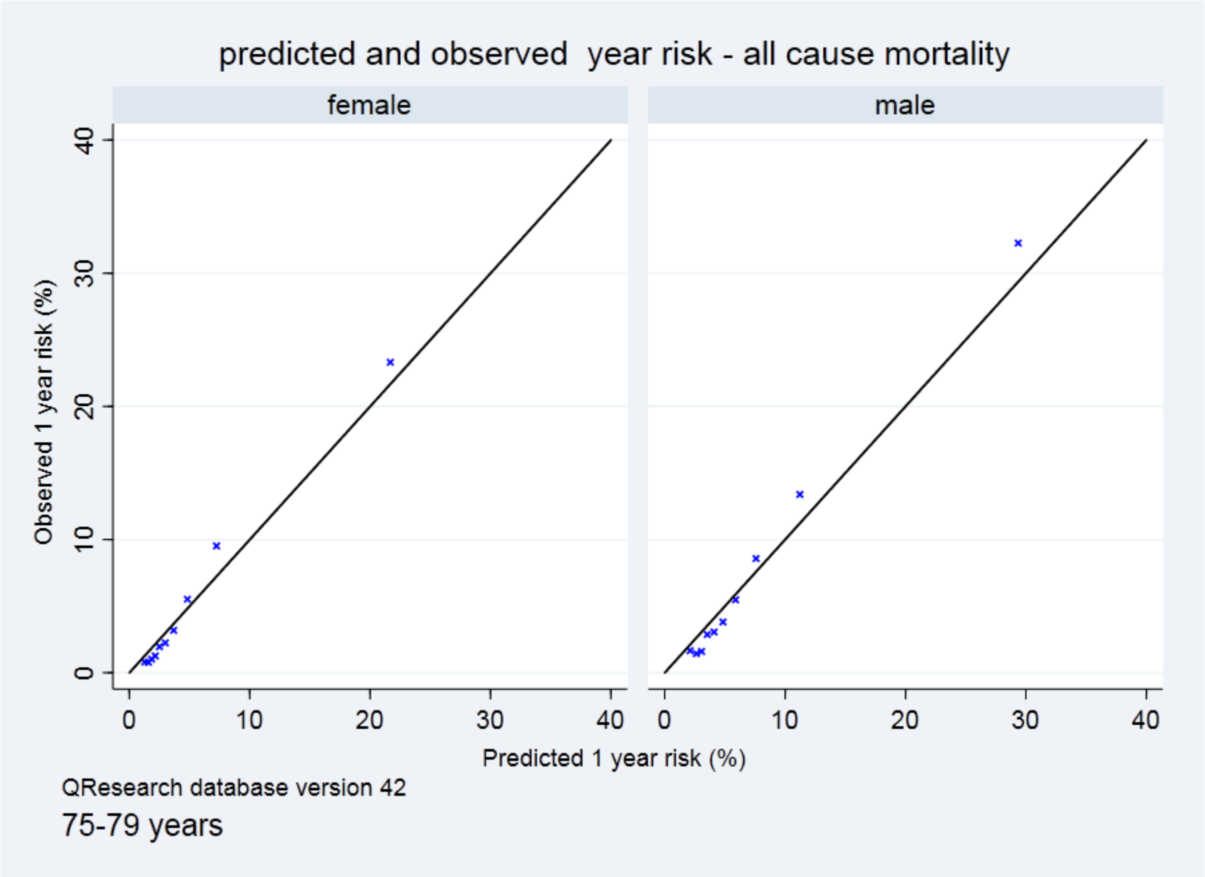

Figure 1d calibration in patients aged 80-84 years

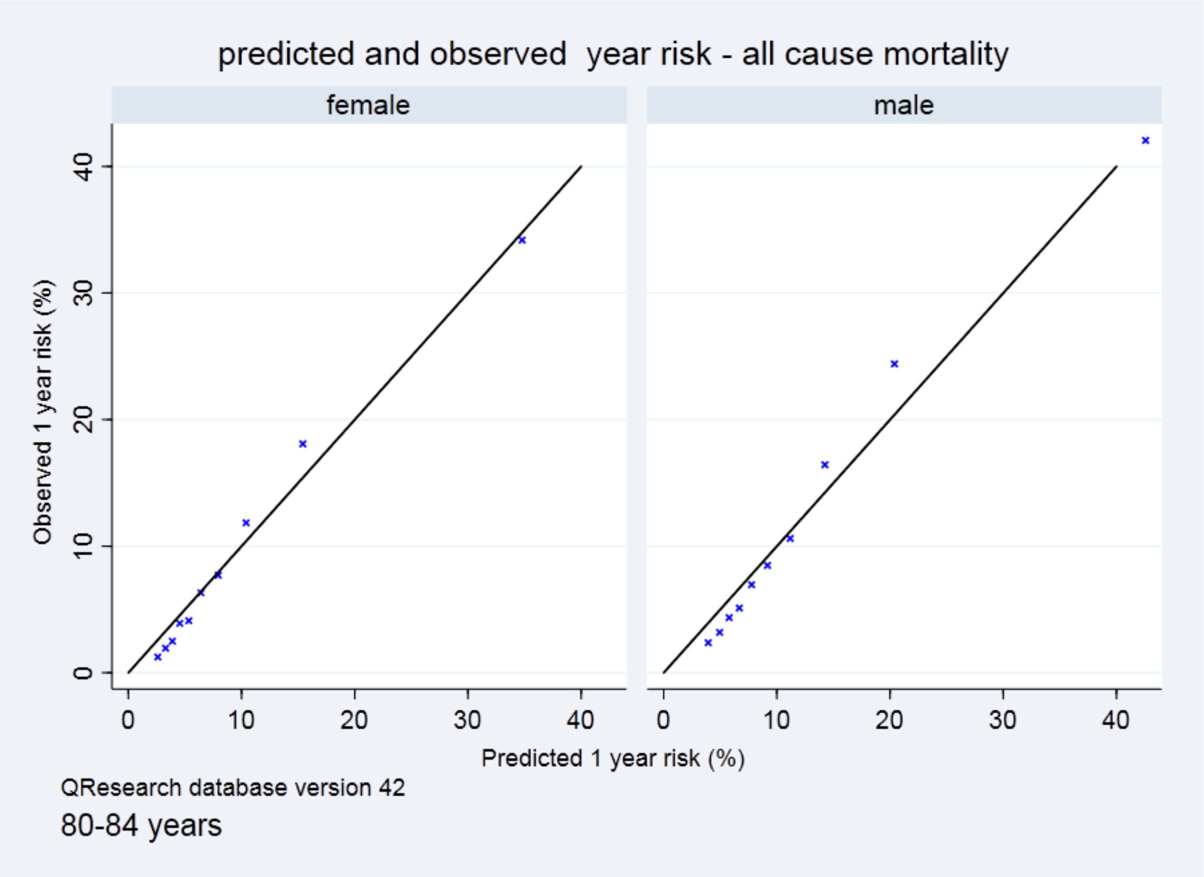

Figure 1e calibration in patients aged 85 plus years

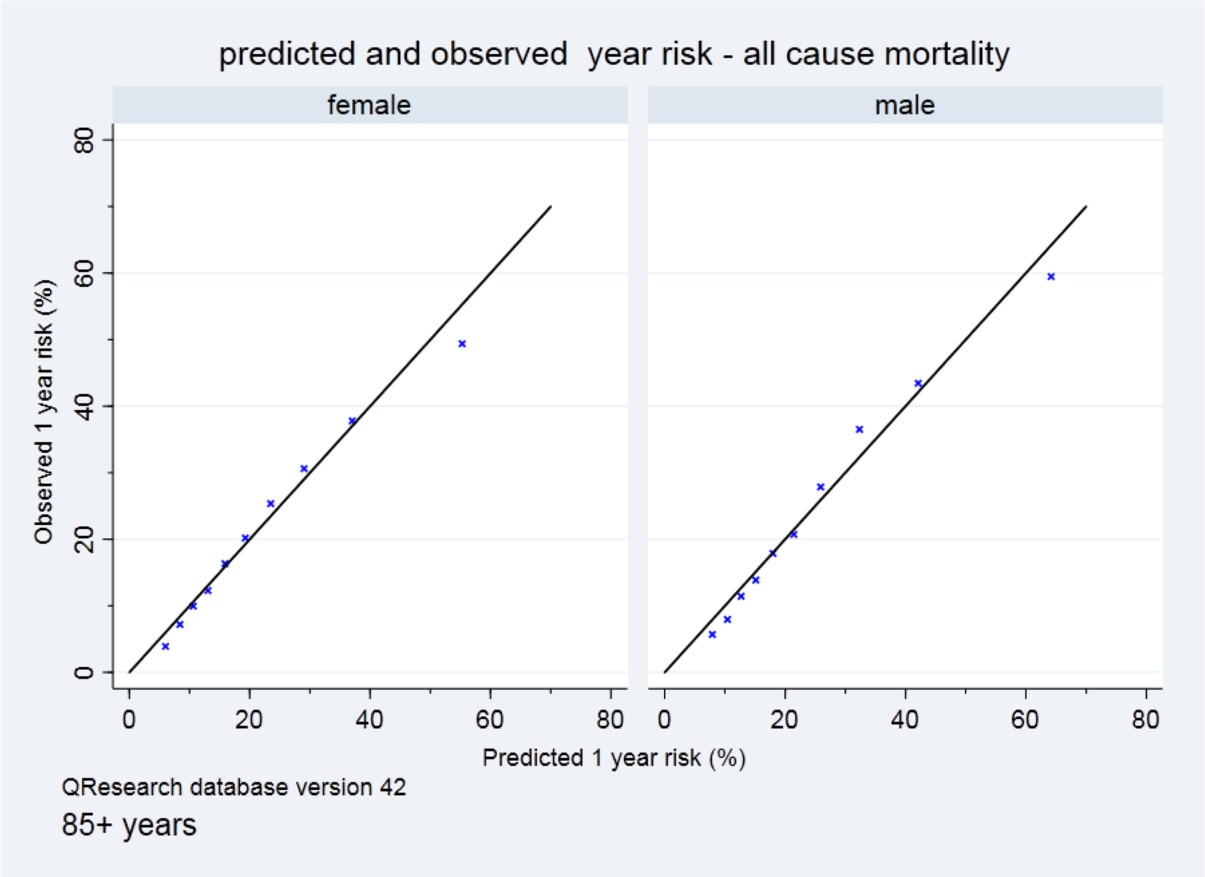

Supplementary tables

Supplementary table 1 number of morbidities in the derivation and validation cohort based on 29 conditions listed in the footnote. Figures are counts (%) unless otherwise indicated.

number of morbidities	derivation	validation
Total patients	1466598	499478
0	253585 (17.3)	86786 (17.4)
1	350994 (23.9)	120596 (24.1)
2	321308 (21.9)	109839 (22.0)
3	233771 (15.9)	79010 (15.8)
4	146557 (10.0)	49596 (9.9)
5	83200 (5.7)	27986 (5.6)
6	43035 (2.9)	14506 (2.9)
7	20346 (1.4)	6657 (1.3)
8	8690 (0.6)	2808 (0.6)
9	3374 (0.2)	1104 (0.2)
10	1162 (0.1)	421 (0.1)
11	397 (0.0)	127 (0.0)
12	133 (0.0)	31 (0.0)
13	34 (0.0)	7 (0.0)
14	9 (0.0)	4 (0.0)
15	2 (0.0)	0 (0.0)
16	0 (0.0)	0 (0.0)
17	0 (0.0)	0 (0.0)
18	0 (0.0)	0 (0.0)
19	1 (0.0)	0 (0.0)

The 29 medical conditions included: atrial fibrillation, cancer, asthma or COPD, CCF, cardiovascular disease, treated hypertension, endocrine disorders, hypothyroidism, renal failure, type 1 diabetes type 2 diabetes, venous thrombo-embolism, epilepsy, leg ulcer, chronic liver disease or chronic pancreatitis, depression, malabsorption , Parkinson's disease, peptic ulcer disease, osteoporotic fracture, diagnosis of osteoporosis , rheumatoid arthritis, learning disability, dementia, manic depression or schizophrenia, deafness, cataract, registered blind or poor vision, falls

Supplementary table 2 performance of the mortality algorithm by age and ethnic group

group	statistic	**women mean (95% CI**	**men mean (95% CI**
65-69 years	D statistic	2.11 (2.04 to 2.18)	1.90 (1.84 to 1.96)
	Harrell's C	.848 (.836 to .86)	.832 (.822 to .843)
	R^2	51.60 (49.98 to 53.23)	46.39 (44.85 to 47.93)
70-74 years	D statistic	2.04 (1.98 to 2.10)	1.84 (1.78 to 1.89)
	Harrell's C	.835 (.824 to .846)	.818 (.809 to .828)
	R^2	49.88 (48.34 to 51.43)	44.59 (43.14 to 46.03)
75-79 years	D statistic	1.85 (1.80 to 1.90)	1.66 (1.61 to 1.70)
	Harrell's C	.811 (.801 to .82)	.792 (.783 to .801)
	R^2	45.02 (43.62 to 46.42)	39.57 (38.23 to 40.92)
80-84 years	D statistic	1.61 (1.57 to 1.65)	1.48 (1.43 to 1.52)
	Harrell's C	.776 (.768 to .784)	.763 (.755 to .772)
	R^2	38.26 (36.98 to 39.54)	34.27 (32.95 to 35.59)
85+ years	D statistic	1.20 (1.18 to 1.23)	1.26 (1.22 to 1.29)
	Harrell's C	.712 (.707 to .718)	.725 (.718 to .731)
	R^2	25.65 (24.84 to 26.46)	27.36 (26.25 to 28.47)
White or not recorded	D statistic	2.28 (2.26 to 2.30)	2.18 (2.16 to 2.20)
	Harrell's C	.852 (.85 to .855)	.844 (.841 to .847)
	R^2	55.45 (55.03 to 55.86)	53.09 (52.61 to 53.56)
Indian	D statistic	2.24 (2.03 to 2.46)	2.26 (2.04 to 2.49)
	Harrell's C	.845 (.812 to .878)	.854 (.824 to .883)
	R^2	54.61 (49.80 to 59.42)	55.00 (50.04 to 59.95)
Pakistani	D statistic	2.04 (1.74 to 2.34)	2.09 (1.84 to 2.34)
	Harrell's C	.817 (.758 to .875)	.829 (.788 to .869)
	R^2	49.85 (42.47 to 57.24)	51.11 (45.13 to 57.10)
Bangladeshi	D statistic	1.83 (1.37 to 2.30)	1.79 (1.46 to 2.12)
	Harrell's C	.800 (.717 to .882)	.798 (.748 to .848)
	R^2	44.49 (31.96 to 57.02)	43.39 (34.40 to 52.37)
Other Asian	D statistic	2.53 (2.11 to 2.94)	2.19 (1.84 to 2.55)
	Harrell's C	.878 (.833 to .923)	.862 (.817 to .907)
	R^2	60.36 (52.46 to 68.26)	53.48 (45.42 to 61.55)
Caribbean	D statistic	2.22 (1.96 to 2.49)	2.11 (1.88 to 2.34)
	Harrell's C	.837 (.8 to .874)	.837 (.804 to .871)

	R^2	54.16 (48.19 to 60.12)	51.51 (46.10 to 56.93)
Black African	D statistic	1.96 (1.31 to 2.61)	1.84 (1.36 to 2.33)
	Harrell's C	.807 (.714 to .9)	.802 (.728 to .876)
	R^2	47.96 (31.44 to 64.47)	44.83 (31.94 to 57.72)
Chinese	D statistic	2.33 (1.69 to 2.96)	2.19 (1.59 to 2.80)
	Harrell's C	.852 (.776 to .927)	.881 (.828 to .934)
	R^2	56.36 (42.97 to 69.75)	53.48 (39.82 to 67.14)
Other ethnic group	D statistic	2.41 (2.14 to 2.69)	2.31 (2.02 to 2.59)
	Harrell's C	.886 (.858 to .915)	.849 (.808 to .889)
	R^2	58.19 (52.59 to 63.79)	55.93 (49.80 to 62.05)

Supplementary table 3 performance of the mortality algorithm in the validation cohort restricted to patients with 2 or more morbidities

		women<br>mean (95% CI)	men<br>mean (95% CI)
	**time period**		
D statistic	1 year	1.99 (1.97 to 2.01)	1.90 (1.88 to 1.93)
Harrell's C	1 year	.818 (.815 to .821)	.811 (.808 to .815)
R^2	1 year	48.48 (47.97 to 48.99)	46.37 (45.78 to 46.95)

Supplementary table 4: sensitivity, specificity, positive and negative predicted value for deaths at different thresholds of risk of death over 1 year in the validation cohort restricted to those with 2 or more morbidities

	risk threshold %	true negative (count)	false negative (count)	false positive (count)	True positive (count)	Sensitivity %	Specificity %	PPV %	NPV %
top 1%	66.2	240,763	48,413	1,146	1,774	3.5	99.5	60.8	83.3
top 5%	40.2	235,319	42,173	6,590	8,014	16.0	97.3	54.9	84.8
top 10%	28.3	227,520	35,367	14,389	14,820	29.5	94.1	50.7	86.5
top 15%	21.4	218,723	29,559	23,186	20,628	41.1	90.4	47.1	88.1
top 20%	16.8	208,962	24,715	32,947	25,472	50.8	86.4	43.6	89.4
top 25%	13.5	198,378	20,694	43,531	29,493	58.8	82.0	40.4	90.6
top 30%	11.0	187,205	17,263	54,704	32,924	65.6	77.4	37.6	91.6
top 35%	9.1	175,529	14,334	66,380	35,853	71.4	72.6	35.1	92.5
top 40%	7.5	163,370	11,888	78,539	38,299	76.3	67.5	32.8	93.2
top 45%	6.2	150,954	9,699	90,955	40,488	80.7	62.4	30.8	94.0
top 50%	5.2	138,131	7,917	103,778	42,270	84.2	57.1	28.9	94.6

Supplementary table 5: Calibration by age, ethnic group and in the top 2%, 10% and 50% of predicted mortality risk

		women					men				
	group	**total**	**deaths**	**observed risk (%)**	**predicted risk (%)**	**ratio of observed to predicted**	**total**	**deaths**	**observed risk (%)**	**predicted risk (%)**	**ratio of observed to predicted**
ethnicity	White or not recorded	261,412	31,850	6.95	7.44	0.93	213,106	27,579	7.29	7.77	0.94
	Indian	3,184	224	4.35	4.90	0.89	2,881	258	5.61	5.89	0.95
	Pakistani	1,599	131	3.95	5.48	0.72	1,561	178	8.01	7.16	1.12
	Bangladeshi	797	55	4.33	4.55	0.95	719	107	10.16	9.19	1.11
	Other Asian	1,444	73	3.14	3.53	0.89	1,287	97	4.74	4.18	1.13
	Caribbean	2,475	173	4.46	5.14	0.87	1,940	239	7.41	7.61	0.97
	Black african	1,294	32	1.36	2.27	0.60	902	60	4.06	4.61	0.88
	Chinese	504	35	4.37	2.43	1.80	403	37	4.31	4.18	1.03
	Other	2,222	153	4.32	4.68	0.92	1,748	165	6.17	6.01	1.03
ageband	65-69	76,772	2,558	1.65	1.56	1.05	74,464	3,465	2.32	2.37	0.98
	70-74	57,903	3,106	2.69	2.69	1.00	52,982	4,302	4.08	4.26	0.96
	75-79	48,750	4,435	4.75	4.95	0.96	41,665	5,460	7.15	7.41	0.97
	80-84	40,746	6,383	8.78	9.43	0.93	29,894	6,228	11.96	12.63	0.95
	85 plus	50,760	16,244	20.69	21.75	0.95	25,542	9,265	23.83	24.96	0.95
**risk group**	top 2%	5,498	3,033	50.58	59.44	0.85	4,490	2,663	56.86	64.63	0.88
	top 10%	27,493	12,457	35.37	36.13	0.98	22,454	10,536	37.68	36.79	1.02
	top 50%	137,465	29,370	13.09	13.47	0.97	112,273	24,878	13.60	13.77	0.99
